# Why art? The role of arts in arts and health

**DOI:** 10.3389/fpsyg.2023.765019

**Published:** 2023-03-22

**Authors:** Björn Vickhoff

**Affiliations:** Clinical Sciences, Sahlgrenska Academy, Gothenburg University, Gothenburg, Sweden

**Keywords:** arts and health, aesthetic contextualism, embodied aesthetics, enactment, imagination/imagery, reuse, sensorimotor empathy, aesthetic empathy

## Abstract

This article is an answer to a report called “What is the evidence on the role of the arts in improving health and well-being?” The authors conclude that the arts have an impact on mental and physical health. Yet, the question of the role of the arts remains unanswered. What is and what is not an art effect? Recently, *embodied* theory has inspired articles on the perception of art. These articles have not yet received attention in the field of Arts and Health. Scholars in psychosomatic medicine have argued for an approach based on recent work in enactive embodied theory to investigate the connection between the body and the mind. The present article examines how key concepts in this theory relate to art. This leads to a discussion of art in terms of empathy—the relation between the internal state of the artist and the internal state of the beholder. I exemplify with a conceptual framework of musical empathy. Implications for health are addressed.

## Introduction

“*It is difficult to say what is meant by art*,

*and especially what is good, useful art*,

*art for the sake of which we might condone such sacrifices*

*as are being offered at its shrine” (Leo Tolstoy)*.

In 2019, the World Health Organization for Europe published a report called “What is the evidence on the role of the arts in improving health and well-being? A scoping review” (Fancourt and Finn, 2019). The report is an evaluation of the field of *Arts and Health*. It covers, directly or indirectly, 3,000 studies. These studies demonstrate wide-ranging effects of a variety of art forms on a variety of health-related variables. Yet, the synthesis question of the *role* of the arts in Arts and Health remains to be answered. This is the main problem in the field. To present evidence of art-effects demands a definition of art. It is a matter of validity. This poses the field to the challenge to define art. Can this be done?

Discussing art definitions, the authors of the report mention cross-cultural characteristics such as value regardless of utility (as expressed in the phrase “arts for art's sake”), imaginative experiences for both the producer and the audience, emotional response, novelty, creativity, originality, specialized skills, and rules of form. The authors conclude that art is difficult to define. The reasoning results in a presentation of a list of art forms. This list comprises:

…performing arts (e.g. activities in the genre of music, dance, theatre, singing and film); visual arts, design and craft (e.g. crafts, design, painting, photography, sculpture and textiles); literature (e.g. writing, reading and attending literary festivals); culture (e.g. going to museums, galleries, art exhibitions, concerts, the theatre, community events, cultural festivals and fairs); and online, digital and electronic arts (e.g. animations, film-making and computer graphics) (Fancourt and Finn, 2019, p. 1).

Few of the art forms on the list meet the cross-cultural criteria, suggested by the authors. Crafts, for example, do not have “value regardless of utility” and are not always creative or original. What is the common property on this list, which justifies the label “art”? The idea of “arts for art's sake” separates art from the beholder. This undermines the idea of Arts and Health. The fact that we, humans of any culture, devote work, time, and costs to art, contradicts that there is nothing in it for us, contradicts that art does not resonate with our lives, contradicts that art is just an object in-its-own-right. Art is not for art's sake. Art is for our sake. The question is “How?”

Studies demonstrating change in health-related variables in art interventions are often taken as evidence of an art-effect. Are they? In studies demonstrating that music is beneficial for brain plasticity (Jäncke, 2009), the effect is attributed to the rich environmental input. The same effect can be seen in mice in well-equipped cages. Are the changes an art effect or a generic effect of stimulating activity? Do studies showing that choir singing has social health benefits (Dingle et al., 2013) indicate an art effect or do they reflect the mere fact that choir singing is a collective activity? Are benefits from going to a museum an art effect or a walking effect? Are increased heart rate variability (Vickhoff et al., 2013) and benefits for breathlessness in COPD patients (Skingley et al., 2014), both caused by the deep respiration in singing, art effects? Is self-esteem following the ability to perform (Franklin, 1992), an art effect? Is the distracting effect of music during medical treatment (Moris and Linos, 2013) an art-effect? Is the cardiovascular effect from dancing (Merom et al., 2016) an art effect? If art is defined by a list of art forms, all these effects are art effects. The evident risk following from the negligence of the validity problem is that the field reports supportive evidence of art effects inevitably compromised by confounding variables. This makes the question of the role of arts legitimate. If the field cannot separate art effects from confounding variables, it cannot answer the question: Why art?

A validation of the art effect requires operational specifications based on a “nominal definition” of art. A nominal definition, as proposed by the 17^th^ century philosopher John Locke, is “an abstract Idea to which the Name is annexed” (Newman, 2007).

Defining art is difficult for several reasons:

1. Art is disparate. Some arts are static, others time varying. Some engage hearing, others sight. Some invite participation, others contemplation.
2. Art is creative. Artists invent new expressions, which do not fit definitions. If there is a recipe, there is no creativity. If it is specified, it cannot surprise. If there is a norm (as in the Soviet Union and in Nazi Germany), it cannot be oppositional.
3. Art has a connotation of quality. A painting is not art just because it is painted. It is a matter of taste and judgment. A benchmark for what qualifies as a work of art is the consensus in the “Art World” (artists, critics and the market). This indicates what is considered art, but does not specify what art is.

Can an overarching theory of Art and Health be formulated? The studies in the WHO-report invoke theories from “psychology, psychiatry, epidemiology, philosophy, ecology, history, health economics, neuroscience, medicine, health geography, public health, anthropology, and sociology, among others” (p. 1). The need of an overarching theory has been highlighted (Koch, 2017; Stickley et al., 2017). Theoretical awareness is important for linking art to health, for research design, for validation of art effects, and for the progress and refinement of art-therapy. In the end, it is beneficial to the patient.

In what sense does art resonate with health? This question connects with psychosomatic health. What is the link between the mind and the body? The *embodied theory of cognition* is such a theory. It has inspired articles on the perception of art (Joy and Sherry Jr, 2003; Freedberg and Gallese, 2007; Calvo-Merino et al., 2008; Xenakis and Arnellos, 2014; Kirsch et al., 2016; Arteaga, 2017; Koch, 2017; Mastandrea et al., 2019; Montero, 2018). Two anthologies have been published: *Embodied Aesthetics* (Scarinzi, 2014) and *Aesthetics and the Embodied Mind: Beyond Art Theory and the Cartesian Mind-Body Dichotomy* (Scarinzi, 2015). Although this massive contribution is relevant to the question of the role of the arts in Arts and Health, embodied aesthetics seems to have slipped under the radar of the field. Kirmayer and Gómez-Carrillo have argued for an approach in psychosomatic medicine “…that builds on recent work in embodied and enactive cognitive science” (Kirmayer and Gómez-Carrillo, 2019).

The application of embodiment to art, is developed in *Embodied Aesthetics* (Ticini et al., 2015). An article called “*The Embodied-Enactive-Interactive Brain: Bridging Neuroscience and Creative Arts Therapies”* has already been presented (Vaisvaser, 2021). This article is however based on the Bayesian approach to cognition and “predictive coding”, which implies a *computational* understanding of the brain. This is at odds with enactive embodied theory.

The aim of this article is to answer the question “Why Art?” The approach is to present and discuss concepts developed in embodied and enactive cognitive science and present a “contextual framework”—“a network of interlinked concepts that together provide a comprehensive understanding of a phenomenon or phenomena” (Jabareen, 2009). I will refer to research in cognitive science, psychology, philosophy and neurology. Considering the complexity of the neurological response to art, it is a giant step to frame these interdependences. We must take into account that there is “still a long way to go before we understand enough about how large numbers of functionally disparate, interconnected neurons generate and use dynamics to control concerted, whole brain activity” (Whittington et al., 2018).

This calls for limitations:

1. Art is discussed in terms of “aesthetical contextualsim” (Levinson, 2007). This is just one of many philosophical art theories. This choice will be discussed and motivated.
2. “Context” is discussed according to “enactive embodied theory”. This choice will be discussed and motivated.
3. The overarching concept “aesthetic empathy” is exemplified with music. This is because music is the most researched art form in neurology. Prerequisites for generalization to other art forms will be addressed.
4. The theoretical level is limited to a “*conceptual framework”*. This is a first stage of theoretical development.
5. There is a lack of precision of concepts due to the semantic problem in the construction of a framework overarching behavioral sciences, neurology and philosophy. We have to settle with explanations of correspondences and correlates.

Outline:

1. *Aesesthetic contextualism*—a philosophical understanding of art based on contextual knowledge in art perception.
2. *The “representation war”*. This is an account for diverging understandings of “context” in the behavioral and brain sciences.
3. *Carving up “context” with ERP*. “Event Related Potentials” provide an entrance for an evidence-based discussion of “context”.
4. *The state of the beholder*.
5. *The state of the artist*.
6. *Aesthetic empathy* understood as the relation between the internal state of the artist and the internal state of the beholder.
7. *A conceptual framework of musical empathy*. The dynamics of key concepts in enactive embodied theory exemplified by a conceptual framework of music. This is followed by a discussion concerning the prerequisites of generalization to other art forms and a framework for static art forms.
8. *Discussion:* How does the presented framework answer the question: Why art?

## Aesthetic contextualism

“Aesthetic contextualism” is one of several directions in the philosophical discussion of art. The concept of “context” includes the state of the beholder in the discussion of art. So does in fact the concept of “aesthetics”. It was adopted by the German philosopher Alexander Gottlieb Baumgarten from the Greek word “aesthesis” referring to qualities of sensation (N.B., the antonym “anesthetic”, as in “anesthesia”, meaning absence of sensation). Baumgarthen suggested that “aesthetics' could be used to mean the sense of beauty and taste (Baumgarten, 1763). Since aesthetics refers to sensations and taste, there is no such thing as an aesthetic object per se. The original understanding of “aesthetics' thus concerns the reaction of the beholder.

Philosophical theories of art tend to follow the art isms (Adajian, 2007). The modernism movement demonstrated that beauty is not necessarily an art property (as can be exemplified with Marcel Duchamp's *Fountain*). Anything could now be presented as art (e.g., Andy Warhol's *Campbell's Soup Cans*, or John Cage's composition *4.33* consisting of pauses and pauses only for 4 min and 33 s). This led on the one hand to a general skepticism about definitions of art, and on the other to all-inclusive, safe definitions, stating that anything presented as art, is art. Versions of this position include: Art is something produced by an artist, art is something exhibited by an art institution, and art is an agreement in the Art World. These definitions are circular. They do not present “an abstract idea to which the name (Art) is annexed”.

The art critic and philosopher Arthur Danto suggested in the 1980s that the unspoken in the work of art, is an art property. He included an *ellipsis* in his definition—an empty space (…) to be filled by the beholder with ‘art historical context”. Danto (1981) argued that the difference between an art object and an everyday object is “not so much the kinds of object as the kinds of attitudes, and hence not *what* we relate to but *how* we relate to it”. With this understanding, Danto downplayed the object in favor of the reaction, which makes his definition applicable to sentient art expressions such as “conceptual art” (which could be anything causing a reaction). Jerrold Levinson suggested the concept of *aesthetic contextualism:*

Contextualism is the thesis that a work of art is an artifact of a particular sort, an object or structure that is the product of human invention at a particular time and place, by a particular individual or individuals, and that that fact has consequences for how one properly experiences, understands, and evaluates works of art (Levinson, 2007).

The word “context” is composed of Latin con: with, and text: textile, suggesting that something is perceived in a weave of information. In cognitive science (i.e., the study of the acquisition and use of knowledge), context means additional information. Additional, that is to the information provided by the object. Abstract art does not move non-artists emotionally as much as artists (Komar and Melamid, 1999; Vartanian and Goel, 2004; Vittorio, 2009), indicating that naïve viewers need to recognize something in the work that they can relate to, or identify with. This expertise/amateur relation to art is paralleled by responses to atonal music (Daynes, 2011). In atonal music, the harmonic context of the melodies is systematically removed. These two studies confirm that the contextual additive creates anticipations, which are crucial in perception.

Contextual information can be:

1. Situational: If we, in the case of Duchamp's *Fountain*, had encountered the work in a bathroom instead of an art gallery, the perception of the object would be different. If we had experienced absence of music, as in the case of Cage's *4.33*, outside a concert hall, we would not perceive it as art.
2. Congenital: Perception is species specific. The information in the DNA double helix expressed in the design of the perception organs, the structure of the body and action possibilities is decisive for perception.
3. Learnt: Individually acquired knowledge. In the case of a work of art, this can be knowledge about the style, as well as personal associations invoked by the work.

Levinson's definition of aesthetic contextualism, is restricted to cultural knowledge. Cultural knowledge is required to identify, understand, and evaluate art, but art is more than this. Art causes physical reactions: tears, goose flesh, accelerated heartbeats, smiles, etc. It can make us feel fear, love, longing, and awe. The perception of art involves commitment, engagement, identification, and acting out (as in the extreme case of “head banging”). In one word—we *live* the artwork with our bodies. The art historian David Freedberg and the neuroscientist Vittorio Gallese has claimed that embodiment is fundamental in the perception of art:

We propose that a crucial element of esthetic response consists of the activation of embodied mechanisms encompassing the simulation of actions, emotions and corporeal sensation… (Freedberg and Gallese, 2007).

Aesthetic contextualism is considered here for the following reasons:

1. Context is mandatory for perception. “No perceptual task takes place in a contextual vacuum” (Abney et al., 2016). Without context, we cannot even perceive a work of art.
2. We cannot expect art to mean something, if it does not relate to the experience we carry into the meeting with the work. The experience of anguish is a prerequisite to the perception of *The Scream* by the Norwegian painter Edvard Munch. The same can be said about the experience of horror for the perception of Edgar Allan Poe's poem *The Raven*, the experience of suffering for the perception of Billie Holliday's interpretation of *Strange Fruit*, and the experience of father-son relations for the perception of Tennessee Williams' play *Cat on a Hot Tin Roof*. Etcetera.
3. Aesthetic contextualism includes the beholder in the understanding of art, which is a prerequisite for the idea of Art & Health. Specifically, the interactive role of the beholder enables an *enactive embodied* understanding of art (Xenakis and Arnellos, 2014).
4. Contextual anticipation has a well-researched psychosomatic effect—the placebo effect (Schwarz et al., 2016).

To present an enactive embodied version of aesthetic contextualism, is to take a stance in the “representation war” (Constant et al., 2020). This war concerns the lack of consensus in the understanding of context in perception. Next, I will make a brief account for the positions in this debate and defend an enactive, embodied approach.

## The representation war

The battle line in the “representation war” is drawn between *computational theory* emanating from Jerry Fodor (Fodor, 1975), and *embodied theory* in the wake of Maurice Merleau-Ponty (Merleau-Ponty, 1945) and James Gibson (Gibson, 1977). “Computationalism” is inspired by the function of computers. Computers can perform several cognitive functions (e.g., memory, calculation, communication, orientation). Acquisition and use of binary information in synapses (spikes) can be modeled with computers. Embodied theory, by contrast, states that the brain can only be understood as a part of a body interacting with its habitat. This has led to separate terminologies. The dividing line appears clearly in the distinction between *predictive coding*, and *embodied anticipation*.

The basic idea in “predictive coding” is that the perception of an environmental parameter results from two distributions of probabilities of information: the expected, and thus contextual outcome and the input (Adams et al., 2013). Applying Bayesian statistics, a third distribution of probability can be calculated, which contains information from both distributions. According to the theory, this third distribution is what we perceive.

Computational theory raises questions:

1. We do not perceive objects as compromises of possibilities, as the Bayesian approach implies. We perceive them as either this or that. If we, to take an example, expect the next tone in a melody to be a C and the played tone is a G, the resulting perception could never be a compromise weighing these two possibilities. This would make music listening unbearable. Music listening requires exact discrimination of pitch relations (Peretz, 2016). In general, the classical bi-stable images in Gestalt Psychology demonstrate that we can only perceive one possibility at a time.
2. The brain is not just hardware and software. “Give a computer the same input and you should get back the same response every time. But give a human brain the same sensory input and you will see a range of different responses. This is because the brain's response to sensory input depends not only on the properties of the input, but also on its own *internal state* at the time when the input is processed” (Iemi et al., 2019) (my italics). The internal state fluctuates due to emotions. Biochemical aspects of emotion (e.g., neurotransmitters) provide the milieu where endogenous neural exchange of information takes place. “Emotions modulate virtually every aspect of cognition” (Tyng et al., 2017). This statement can be reversed; cognition modulates emotion (Ochsner and Gross, 2005). A theory of perception thus must include emotion. Emotion is as much a body state as a brain state.
3. Computers do not oscillate. Brains do. The research of neural oscillations has revolutionized the behavioral and brain sciences. This is accompanied by theoretical development such as Neural Resonance Theory (Large, 2008) for music perception and the Binding by Synchrony Hypothesis (Singer, 2007), suggesting that synchronized oscillations bind distributed brain activity.
4. Is “code” a useful concept? “Predictive coding” presumes that the brain makes inferences from its own coded activity. This issue is called the “symbol grounding problem”, which reads: “How can the interpretation of a symbol system be made intrinsic to the system?” (Harnad, 1990). If the code cannot even theoretically be decoded, it is not a meaningful concept. On these grounds, the code-metaphor, cemented in neurology as it is, has been questioned (Brette, 2019). This is however not self-evident. It is hard to deny that the environment is “encoded” (since environmental information is transformed into electric neural activity) and it is hard to deny that neural activity is “decoded” (since we do perceive the environment). The problem with the code-metaphor is that it easily presumes an interpreter, which leaves us with the problem to find a homunculus in the brain. The solution may be that the “code key” is intrinsic to the system. It is arguable that the key is DNA-information converting action to perception and perception to action, and thus that every living organism and every cell in this organism has access to the “key”.

Embodied theory emanates from Merleau-Ponty's phenomenological theory of perception. It involves the physical attributes of the body and bodily skills in the perception of objects and events (Merleau-Ponty, 1945). The fit between the ability of the body and the environment results in an *intention* to act. The understanding is however not that we perceive the environment and decide to act on it, but that perception and action are interwoven. They are two sides of the same coin: *perception-action*. Mingers, considering the smallest unit of life—the cell (e.g., an amoeba or a neuron)—states that it has “…both a sensory and an effector surface” (Mingers, 1991). This makes perception-action an undividable biological unit. The one cannot exist without the other (e.g., seeing and the accommodation of the eye-lens). This is congenital, but just to a certain extent. Perception-action is also learnt. The exploration of the world by reaching, touching and tasting is typical for babies. This coordinates motor activity with sensory feedback. Viewing a cup is to actualize earlier experiences of reaching for it, grabbing it, lifting it, holding it to our lips, drinking from it and so forth. This is called perceptual learning (Gibson, 1969). If we consider DNA as the “code key”, we learn how to use this key. Perception-action is embedded in the organism at every level. It unites the organism with the world. Gibson (1979) suggested the concept of *affordance*, to denote that an object affords action possibilities, given the design of the body. When we perceive an object, we automatically actualize action opportunities. Gibson specifically pointed out that the affordance “cuts across the dichotomy of subjective-objective” (Gibson, 1979, p. 4).

The beauty with the “affordance” concept is that is unites cognition with emotion. Environmental information concerning food is an affordance, only if we feel hunger. If we are hungry, we tend to detect food. This is reciprocal. If we smell food, we tend to get hungry. This illustrates the cognitional-emotional interdependency.

Perception-action penetrates the border between the organism and its environment, allowing for an analysis of cognition as an information system, consisting of interacting subsystems (e.g., the organism, other agents, surroundings).

Gibson proposed that “the perception apparatus” “tunes in” to the environment. He described the fit of environmental and endogenous information as “resonance”:

Instead of supposing that the brain constructs or computes the objective information from a kaleidoscopic inflow of sensations, we may suppose the orienting of the organs of perception is governed by the brain so that the whole system of input and output resonates to the external information (Gibson, 1966, p. 5).

Gibson treated “resonance” and “attunement” as two complementary mechanisms. He claimed that a “perceiver is a self-tuning system” and that the development of this attunement, or “the education of attention, depends on past experience but not on the storage of past experiences” (Gibson, 1979). The phrase “education of attention” suggests that we learn how to perceive (i.e., “perceptual learning”). The phrase stating that this “…depends on past experience but not on the storage of past experiences” proposes that experiences leave traces in the nervous system, but that this does not entail that memories are stored in the brain like books in a library.

Gibson's theory is still relevant in contemporary debate and research. “Resonance”, “attunement” and “pick-up” are however metaphors. The more exact understanding of these concepts has occupied the behavioral and brain sciences since.

There are two schools of embodied theory. “Enactive theory” is contextual, whereas “radical embodied theory” claims *direct perception*, which means that all the information in perception is environmental. Chemero argues that it follows from Gibson's ecological approach to perception that perception “does not involve computation or mental representations”, and that perception “is primarily for the guidance of action, and not for action-neutral information-gathering”. He draws the conclusion that since “perception does not involve mental addition of information to stimuli, yet is able to guide behavior adaptively, *all the information* necessary for guiding adaptive behavior must be available in the environment to be perceived” (Chemero, 2013) (my italics). This cannot be concluded from Gibson's theory, since the resonance metaphor suggests a fit of two variables of information. Action possibilities are constrained by species-specific information as well as individually acquired knowledge how to move (skill). This is also the position of Merleau-Ponty who emphasized that perception depends on physical attributes of the body and bodily skills. Merleau-Ponty labeled action possibilities “the body schema” (Merleau-Ponty, 1945). The body schema is thus contextual information in perception-action. This does not require awareness.

Chemero's position is contradictory to consensus in cognitive science. “No perceptual task takes place in a contextual vacuum” (Abney et al., 2016) 2016. Astonishingly, Chemero is co-author of Abney's article, implying that he does not see context as additional information. The article exemplifies contextual influence with the perception of bi-stable illusions, showing that it is hard to perceive objects if we do not know what to look for. Anthony Norcia's “Coffer Illusion” demonstrates that circles in a presented picture cannot be perceived if we do not know that there are circles to be found. Even more striking is Daniel Simmons' “monkey business illusion” (Simons, 2010). Simons presented a video showing people passing a ball between them. Viewers were asked to count how many times the ball is passed. In this video, someone dressed as a gorilla enters the scene, steps up in front of the camera and salutes the viewers with a drum vortex against her chest. Still, 50 % of the viewers do not see the gorilla if they are not informed that a gorilla will appear. Furthermore, the classical ambiguous pictures in Gestalt psychology, illustrate that we can control cognitively what we see.

What then do radical cognitivists claim? Raja states: “The basic framework of direct perception is that there are high-order variables that specify the features of the environment and that perceivers detect them without any kind of cognitive load or information processing (Raja, 2019). As an example, Raja proposes a literal understanding of Gibson's concept of resonance and compares perception with the resonance of musical instruments. This implies that the nervous system resonates to environmental features just as the strings in a piano resonate to a voice singing into it. “Non-linear resonance may be the way perceptual systems self-modulate and are modulated in their relation to perceptual information, thus being able to exhibit changes in the detection of such information” (Raja, 2018). Here, Raja refers to Neural Resonance Theory (Large, 2008).

Raja's statement is self-contradictory. A variable, “higher order” or not, *is* information. Resonance concerns the fit between two variables of information. This claim has been proved in original research explaining resonance with information theory (Goychuk and Hänggi, 2000; Rosso and Masoller, 2009). Information system theorists point out that it is important to note that “…an affordance arises from the user/artifact relation, not just from the artifact” (Fromm et al., 2020). Furthermore, “self-modulation” implies learning. Learning concerns acquisition of information. The nervous system *learns* how to resonate with environmental rhythms by means of plasticity (Tichko et al., 2022).

*Enactive theory* is a description of the interplay between the subjective and the objective:

We propose as a name enactive to emphasize the growing conviction that cognition is not the representation of a pregiven world by a pregiven mind but is rather the enactment of a world and a mind on the basis of a history of the variety of actions that a being in the world perform (Varela et al., 2016, p. 9).

Enactivists argue that organisms know how to interact with the environment through the history of the species interaction in its domain and by individual learning (Maturana and Varela, 2012). Varela and Maturana described the interdependence between the organism and the surrounding as a *structural coupling*. Structure, contrary to entropy, is selected information. Structural coupling thus concerns a fit of information. Mingers exemplifies with light-sensitive neurons that “it is the structure itself that determines what can be a trigger for it” (Mingers, 1991). The same is obvious in the case of piano strings. Gibson's resonance-concept suggests a structural coupling, but structural coupling is the wider concept. In both cases, perception concerns a fit between environmental information and endogenous information. Just as the “affordance”, structural coupling cuts through the subjective and the objective.

To conclude: In spite of the progress in neurology, the “representation war” reveals that, there is no consensus in the understanding of perception. The terminology is metaphorical (e.g., “codes”, “computations”, representations”, “processing”, and “resonance”, “attunements”, “affordances” respectively). I strongly suspect that differences in this “war” is due to differences in the understandings of the metaphors. In order to come to an armistice, the terminology needs to be specified. This requires an evidence-based discussion. Specifically, we need to carve up the term “context” into an operational understanding. Neurological research of *Event Related Potentials* (ERPs) offers an entrance to this discussion.

## Carving up context with ERP

EEG is a conglomerate of electrical activity emanating from neural oscillations generated in single neurons, pairs of neurons, and fields of neurons. The EEG consist of components, associated to states, behaviors and tasks. Violations of expectancies, so called “prediction errors” are detected as changes in EEG potentials. These brain reactions are called “event related potentials” (ERPs). The ERP reaction indicates a conflict between endogenous (i.e., contextual information) and exogenous information. This is an indirect way to assess context. Reaction times on violations differentiate between the components of expectancies. Although ERPs initially were found for visual surprises (Squires et al., 1975), it is an excellent approach to investigate expectations in music.

A specific ERP, the “early right-anterior negativity” (ERAN), has been detected for tones which do not fit into harmonic context (Koelsch et al., 2018). Interestingly, this reaction is detected not just in the brain of the listener but also brain of the musician, even if the “error” is deliberately planted. This demonstrates that although the musician has an accurate expectancy, her brain reacts as if surprised. The authors interpret ERAN as a violation of “musical syntax”, arguing that the perception of a tone out of harmonic context can be compared to the perception of a word misplaced in a grammatical context. Following Neural Resonance theory, it is however conceivable, that ERAN indicates a mismatch of oscillations.

Music is a cultural application of universal laws of acoustics. In short, the timbre of a played tone is defined by the overtone series (the harmonics). The scales used in music in all cultures can be derived from this overtone series (Large et al., 2016; Kim and Large, 2019). The overtone series consists of resonance relations. Melodies and harmonies are made up from these relations.

Neural Resonance Theory states that music perception should be analyzed as resonance between exogenous oscillations (the soundwave) and endogenous oscillators (neural) according to nonlinear dynamic systems theory (Large and Kolen, 1994). This is fully developed in (Large, 2008). The theory requires that chemo-electrical biological systems can be regarded as physical systems, allowing for physical laws of resonance to be applied to the nervous system. Large describes how excitatory/inhibitory neural ensembles sustain oscillation and knits this to the structures of the hearing apparatus such as the cochlea and the auditory cortex (Large, 2010). One to one resonance between exogenous oscillations and endogenous oscillations explains the perception of musical beat (Large et al., 2015). Large and colleagues also model “mode-locking”, which concerns how the harmonics in one wave locks to harmonics in another, such as the prime to the octave, the prime to the fifth, the fifth to the third etc. This cross-frequency locking is relevant to the perception of tonality (Large et al., 2016) as well as the perception of consonance/dissonance (Kim and Large, 2019). According to the theory, tones outside the harmony cause a clash in resonance between exogenous oscillations and endogenous oscillations. A prerequisite for oscillatory explanation of dissonance is that pitch differences can be discriminated with high precision. Humans can differentiate pitch differences down to 0.2%. Single neurons cannot oscillate fast enough for this precision. This requires a subsequent temporal analysis. In a review of oscillations in the auditory system (Bahmer and Gupta, 2018), the authors show that “…the ability of humans to distinguish changes in pitch can be explained as a precise analysis of temporal information in auditory signals by neural oscillations”.

Neural Resonance Theory claims lawful activity in perception. Dubois differentiates between two kinds of anticipation. Anticipation is “strong” when it is “not based on a prediction from a model of the physical system but is embedded in the fundamental system” (Dubois, 2000). “Weak anticipation”, by contrast, is based on a model. Weak anticipation does not entail that this model is stored as an available representative memory in the brain, but as an simulation (Stepp and Turvey, 2010). In terms of neural activity, calling to mind “…involves reactivation of traces left by perceiving” (Anderson, 2014, p. 96). This implies that weak anticipation requires learning. The recall is an imagination caused by a simulated perception in reused traces.

Violations of strong anticipations in music can according to Neural Resonance Theory be exemplified with a change of rhythm, a change of tonality and dissonances (i.e., purely physical violations), whereas violations of weak anticipations are caused by deviations in musical styles. A violation of a strong anticipation would always elicit a neural reaction, irrespective of mental expectation, since it is a law bound mechanism. In other words, it does not matter if the musician knows that she is playing outside the harmony, there is still a clash in resonance and the brain reacts, as indicated by ERAN. The violation is felt, not just by the listener, but also by the musician. This is because mismatches are arousing. The unexpected move in music (e.g., change of melody, tonality, volume, harmony, instrumentation, rhythmical accents) is accompanied with physiological reactions—which can be assessed as pilo-erection (gooseflesh), accelerated heart rate, diminished heart rate variability, accelerated respiration, diminished finger temperature, and increased skin conductance, indicating arousal (Vickhoff et al., 2012). The arousal calls attention to the unexpected event.

The planted surprise is common in music. In jazz, this can be exemplified with the “in-out” technique, signifying that the soloist plays alternately inside and outside the harmony given by the succession of chords. Notably, soloists on such wild excursions often lean sideways as if stepping out of the body (where the anticipation resides) and into unpredictable territory.

The ERAN reaction is fast. This suggests that violations of strong anticipations are less time-consuming than violations of weak anticipations. Reaction times also separates unattended reactions (fast) from attended reactions (slower) (Rohaut and Naccache, 2017). This confirms the preconscious side to perception. It leads to the question: How can we predict, if we are not aware of what to expect? Dubois showed that biological systems can anticipate without awareness. He modeled strong anticipation mathematically by the mechanism of “delay coupling”. This implies “coupled systems of previous sections where a delayed system is synchronized with the non-delayed version of itself, i.e., a coupling arrangement” (Dubois, 2000). This “anticipation” is not a subjective hunch that something will happen, neither does it require a predicting homunculus in the brain. In Dubois' understanding, “anticipation” is rather a literal translation of Latin “anticipates”, from ante (pre) + cipare (take). In the following, I will call this the “pre-take” to avoid confusion with subjective anticipation. The nervous system pre-takes a state to the effect that we perceive moving objects where they actually are, which would be impossible due to neural delays. This can be associated to Nijhawan's “motion extrapolation in catching” (Nijhawan, 1994), later referred to as the “flash lag” effect for the visual system (Nijhawan, 2002).

How do we coordinate our movements with environmental events, such as Djokovic's return of tennis serve, the peregrine's catch of a starling, or the musician performing in rhythmic synchrony with an ensemble? A first requirement is that objects are perceived where they actually are (in spite of neural delays). A second requirement is that our own movement must be initiated ahead of the event. Indeed, ERP-studies demonstrate not just a post-stimulus reaction, but also a pre-stimulus reaction on events presented in a sequence (Niemi and Näätänen, 1981). Predictive timing involves a hierarchical network of oscillators (Arnal et al., 2011; van Ede et al., 2011).

Coordination and timing is important, not just for sports and music, but for every situation in life. Dubois offers an approach to this so far unsolved problem. His theory of anticipation goes hand in hand with Neural Resonance Theory. Original research reveals that external periodical sounds produce bursts of neural gamma oscillations on sound onsets. These bursts continue when the stimulus is omitted (Snyder and Large, 2005). The burst on the omitted sound is a pre-take.

Mismatches can be attended, or not. The study of “amusics” in ERP research illuminates this distinction. Amusics are people who are unable to perceive music as music, due to congenital or achieved impairment (Peretz et al., 1998). “Amusics fail to detect ‘wrong notes' that violate pitch regularities of Western music” (Peretz, 2016). ERP studies indicate that amusics react on pitch violations (measured as “mismatch negativity”), just as accurately as control groups do (Moreau et al., 2013). The groups however deviate in ERP “positivity” (P300/P600) which signifies awareness. Amusics do not have this reaction. This made Peretz conclude: “The core deficit in congenital amusia resides in a lack of conscious access to processed pitch deviances” (Peretz, 2016). There is a mismatch, and the brain reacts to it, but the listener is not notified. An even more striking example of non-attended brain activity in music perception, is provided by the finding that amusics, in spite of their inability of conscious discrimination of pitch, can imitate melodies (Hutchins and Peretz, 2012). This underlines that perception-action is a pre-conscious undividable biological unit. Interestingly, “amusics” are emotionally affected by music. They can account for these feelings just as well as other people can (Peretz et al., 1998). Meaning demands consciousness but meaning is not always required for emotional experiences—feelings. Meaning can be a consequence of feelings. If an activity feels right, we tend to come up with arguments for that activity—rationalizations.

ERP-reaction times reflect phylogeny (Scheumann et al., 2017). The reaction times indicate that perception-action is the original building block of cognition and that this has been “embellished” with awareness later. The snake bites. This does not demand awareness. It is DNA information in action. It is reptile brain function.

ERP research confirms enactive theory. Coupling between the organism and the environment is indirectly demonstrated, since ERPs are reactions of the opposite. This involves structural coupling since a structure is constrained information. This understanding is consistent with Neural Resonance Theory, which concerns resonance between two variables of information: the structure of the exogenous wave and the structure of the endogenous wave. Coupling implies synchronization of endogenous and exogenous sequences of information; mismatch is desynchronization.

There is a debate concerning the neural underpinning of the ERP-reaction. The *phase reset model* (Sauseng et al., 2007) claims that the reaction reflects a change in background neural oscillation. This has been demonstrated by original research (Mormann et al., 2005; Rizzuto et al., 2006; Jutras et al., 2013; Woolnough et al., 2022). New evidence …”provide strong support for the unification of neuronal oscillations and evoked responses (Studenova et al., 2021). Raja and Anderson suggest that the reset is a bifurcation in a meta-stable system (Raja and Anderson, 2019), where the meta-stable system is the pre-stimulus internal state. In dynamic systems theory, a “bifurcation” is a small manipulation causing dramatic qualitative change in the system.

The reactivation of brain areas for memory tasks indicate that each type of memory can be described as an “alliance of brain regions” connected by “frequency-specific patterns of interregional phase synchronization in large-scale networks” (Dudai and Morris, 2013). The authors claim that these patterns provide multiple contextual information concerning a passed event. This makes the ERP reaction an important learning factor (Tseng et al., 2007). Mismatches dissolve these networks by way of desynchronization and reconnect to them to new alliances by way of synchronization. This is in accordance with the *Binding by Synchrony Thesis* (Singer, 2007), claiming that neural oscillatory synchronization binds distributed activity. Singer's thesis is confirmed by original research. Cortical networks are connected by synchronized brain oscillations (Düzel et al., 2010; Bechtel, 2013). This makes neural networks temporary. They are established through synchronization and fall apart when desynchronized. Repeated confirmation of matches, strengthens the connections in the network (Hebb, 1949). Repeated mismatches dissolve them.

To sum up:

1. The ERP reaction signifies a mismatch between an environmental event and the pre-take.
2. The pre-take synchronizes the organism with environmental sequences.
3. The pre-take is an enactment.
4. The pre-take requires endogenous information. This information is contextual.
5. The ERP reaction is an inverse index of structural coupling.
6. The ERP reaction signifies an instant change of context.
7. Short reaction times indicate violations on “strong anticipations”. This is just a misfit of information. Consciousness about the expectation does not interfere with the reaction. Even so, the reaction can be felt.
8. Longer reaction times indicate “weak anticipations”. This reaction concerns misfits of learnt models (i.e., simulations) and environmental information.
9. Since weak anticipation is based on a model, it is a representation, and if attended, it is a mental representation.

ERP research shows that perception always depends on endogenous activity constituting information, such as the structure of brain waves. This contextual information can be representative or not, attended or not. Cognition is reciprocally dependent on endogenous and exogenous information.

The ERP technique provides a possibility to operationalize context, since it is an index of contextual mismatches. Context can thus be assessed as missing components in ERP.

This understanding of context is relevant for an updated understanding of “aesthetic contextualism”. I will proceed by discussing art as the relation of endogenous activity of the artist and the endogenous activity of the beholder—as empathy. This demands an encircling of the state of the beholder at the moment of art perception and an encircling of the state of the artist at the moment of creation (or performance).

## The state of the beholder

“Aesthetic contextualim” implies that the information in the work of art is related to a weave of information at perception. This is the cognitive side of perception. Cognition and emotion are however interdependent. In order to discuss the perception of art, we must consider the *state* of the beholder—the total being, the embodied mind, including memories, emotions and the conception of the self.

Enactive embodied theory implies that memory is a neural simulation of the activity at previous perception-actions. Traces from previous perception-actions are according to the Hebbian plasticity rule strengthened by repeated use (Hebb, 1949). The state of the beholder of art is continuously changing, as aspects of the work of art activate these paved traces.

I will make a brief account of behavioral aspects of memory and argue that all memories are embodied and emotional. The purpose is to demonstrate that enactive embodied theory is valid for the perception of art.

*Associative memory* is “the ability of living organisms to perceive contingency relations between events…it allows anticipation of an event on the basis of another” (Jozefowiez, 2012). This is the brick of learning. Memory, even at this basic level, is anticipative and thus contextual. Examples of associative learning are classical (Pavlov, 1910) and operant (Skinner, 1963) conditioning. As demonstrated by Pavlov, the ring of a bell can be associated to food. He measured this association as salivation in dogs. This experiment illustrates that perception-action is a learnt application of a congenital capacity. The bell becomes an affordance, as indicated by salivation. Associative learning appears at all levels in the animal realm. Even insects are capable of associative learning (Polilov et al., 2019). A design for temporal sequence learning based on associative learning has been presented (Bose et al., 2005), suggesting that associative memory is the building block of sequenced memories. It is however not enough for the timing of the events in a sequence, just for the order of events.

Associative learning is relevant for the perception of art. The bell in Pavlov's experiment is an arbitrary sign (i.e., there is nothing connecting the sound of the bell with food before the association is made). The bell could be replaced by the word “food”. This makes the sound of the bell a *symbol* for food, rather than an iconic or an indexical sign in Charles Sanders Pierce's sign system (Misak, 2004). This extends embodiment of art to the rich domain of symbols. Words, sentences, poems, novels, lyrics in songs and notes on a sheet of music are embodied through associative learning. We strive with the mountaineer Hillary in “High Adventure”, drink with Hemmingway in “The Sun Also Rises”, feel the humiliation of the characters in Steinbeck's “Mice and Men”, and are sexually aroused by Anaïs Nin's “Delta of Venus.” Pavlov's experiment could concern indications of sexual arousal to Nin's text, just as well as salivation to the bell. This makes Nin's text an affordance.

The British philosopher Gilbert Ryle made a distinction between *declarative knowledge* as the knowledge of *what*) and *procedural knowledge* (the knowledge of *how*) (Ryle, 2009). The former concerns facts presented as symbolic representations and the latter *skill*—knowledge of how to perform an action, such as riding a bike. Procedural knowledge is *implicit*—knowledge that cannot be accounted for. It is learnt by trial and error (plasticity and prediction errors).

Skill is an everyday connotation of art. We perceive the skill of artists as manifested in their works—as a quality. Skill is not just about the ability to perform; it is also essential in perception. Sports commentators recruit former athletes as sidekicks. Athletes know how to perform technically and see details others cannot see. It follows from the mutual interdependency in perception-action (and makes sense), that we feel the skill of the artist through our own skill. We perceive dancing through our own ability to move, melodies through our own ability to sing, etc. This is in accordance with the finding that action observation “activates premotor and parietal areas in a somatotopic manner” (Buccino et al., 2001). Buccino concludes that the observation of an activity automatically activates the same sensorimotor network as when we perform the activity ourselves, even if we do not move. Viewing someone do something is a simulation of the act—an enactment. Skill, in terms of action-possibilities, is contextual in perception. The research of the importance of skill in art perception, is still in an initial state. It indicates that the skill of the beholder is correlated to a positive response to the work (Kirsch et al., 2016).

Skill is obviously embodied knowledge, but so are symbolic representations. As concluded from Pavlov's experiment, it does not matter if the dog is presented with food or a symbol for food, once the association is made.

*Perceptual learning* (Gibson, 1969) is closely related to skill, since skill is mandatory to perception. Perceptual learning can be exemplified with the “reversed goggles” experiment, where the subject learns to navigate although the inflow of visual information is reversed (Harris, 1965). Recently (Tichko et al., 2022) presented a framework demonstrating how an endogenous motor planning oscillatory network learns to perceive exogenous rhythmic patterns. The authors connect this learning to James Gibson's concept “attunement”, suggesting that the organism learns how to “tune in” to the external source. Perceptual learning is a developmental process combining short-term plasticity and long-term plasticity (corresponding to short-term memory and long-term memory). According to the authors, plasticity can be fast enough for a child to learn to entrain a rhythm while listening.

*Episodic memories*, are “retrieved personal experiences and their temporal relations” (Tulving, 1972). Episodic memory implies an activation of motor areas (Nilsson et al., 2000). It is thus a replay of a sequenced event involving motor activity. This makes it related to skill. The recall of a song is an episodic memory, since it is a retrieval of a personal experience replayed by the brain with the temporal relations intact.

*Autobiographical memories* are memories connected to an individual's personal history. They make up the sense of who we are and are thus of existential importance. Autobiographical memories are closely related to episodic memories (Conway, 2001; Gilboa, 2004), although autobiographical memory is the wider concept. Familiar music triggers autobiographical memories. The replayed sequence opens a window to the past. Music has marvelous effects on demented people (Sachs, 2008). Demented residents in elderly care, totally locked into themselves, starts moving when listening to music, suddenly expressing themselves emotionally. They light up, and when asked, they can give a detailed account of when and where they first heard the music, the name of the band, the name of their dancing partner etc. Perceiving music as a composition by Pat Metheny, involves an actualization of going to a concert and enjoy music in company with lovers of jazz guitar music. It entails the identification of the self as a member of this tribe of good taste and skill. The music psychologist John Booth Davies coined the autobiographical effect “Darling, they are playing our tune” (Davies, 1978). This is beautifully illustrated with the song *As times goes by* in the classical film *Casablanca* (1942), including Ingrid Bergman's classical line: “Play it again, Sam!”

Autobiographical memory engages a broad neural network (Bréchet et al., 2021). The mnemonic power of music is well-researched (Yalch, 1991; Wallace, 1994; Rainey and Larsen, 2002; Moussard et al., 2014; Thaut et al., 2014). Thaut's study provides evidence that music induces “oscillatory synchrony in neural networks underlying memory”. It has been shown that music listening enhances synchronization in the alpha2 band, demonstrating an increase of functional connectivity (Wu et al., 2013). External auditory rhythms stimulate memory in patients with Alzheimer's disease (Clements-Cortes et al., 2016). This effect can also be achieved by transcranial rhythmic stimulation (Bréchet et al., 2021). The research indicates that exogenously induced rhythm synchronizes and connects brain areas.

Taken together, these studies suggest that the mnemonic power of music depends not just on associative memory but also on brain resonance to musical rhythms. It is however not likely that the neural frequency connecting brain areas complies with the rhythm in the specific piece of music at hand. There are nonetheless three factors to consider:

1. Musical rhythms include a broad spectrum of subdivisions of the beat, played by the drummer as well as other instrumentalists (performed as ¼ notes, 1/8 notes, 1/16 notes etc.).
2. Cross-frequency coupling, which means that harmonics of the beat resonate with brain oscillations.
3. Perfect conformity between frequencies is not required for resonance. Brain resonance to a tone is a so called “Arnold tongue” with an “envelope” centered around the pitch.

Music can cue to memories, just as other things or events can by means of associative learning, but the reason why music is so effective is the rhythm “massaging” associative connections.

*Emotional memories* are memories connected to emotions. Since all memories are more or less emotional, this is rather an aspect of memory than a category. The word “emotion” consist of “em” (within) and “motion”, i.e., to be moved from within. The mere word reflects the human experience that emotions are bodily displayed. (Hajcak et al., 2007) showed that emotional pictures presented to subjects affect the motor evoked potential in the supplementary motor area (SMA). SMA and the pre-SMA are motor planning areas. In a review article on SMA function (Lima et al., 2016), the authors specify that the border between the pre-SMA and the SMA has been repeatedly associated with emotional music and emotional prosody. “Emotional prosody” refers to the observation that the same sentence can be delivered in different ways, depending on the emotion of the speaker. Since music as well as prosody is produced through motor activity, both contain information concerning a motor sequence. The research on the role of SMA/pre-SMA demonstrate reciprocal motor-emotion dependencies.

This review exemplifies two reciprocal dependencies: motor activity and emotion, and motor activity and memory. This supports embodied cognition theory.

The understanding that a memory is a reactivation of a neural network involving motor function is expressed in *The Sensorimotor Model of Memory*. The model states that…

“…a given event fundamentally consists in perceptual information so that a reactivation of the same sensorimotor circuitry originally involved in its perception is also at stake whenever the event is recalled or comes to mind. In this respect, remembering is tantamount to creating mental simulations of bodily experiences in modality-specific regions of the brain. Memory consists in partial (or covert) reenactments of sensory, motor, and introspective states…” (Ianì, 2019).

This suggests that calling to mind is an imagination, based on a simulation. The Sensorimotor Model of Memory predicts that the neural activity at recall is correlated with the neural activity at perception. Ianì reports evidence for three kinds of correlated motor activity at recall and perception:

1. Movements connected to emotions, such as gestures, facial expressions, prosody and music)
2. Motor activity in perception-action, such as eye movements and touching (or, very convincing, the phenomenon that my dog starts waving his back leg, when scratched behind the ear)
3. Implicit motor simulation of the movements of an observed person.

It follows that motor activity is a mnemonic to experienced events (since motor activity is crucial in the alliance of brain functions involved in memory). This enables an activation of a wide context including autobiographical memories.

Discussing the mnemonic power of motor activity, it is informative to consider the role of the sequence. Just as the sequence of notes is decisive for the flowing hands of a pianist, the brain sequences action patterns over time. We can only retrieve a sequence in the learned direction. The difference between recalling a sequence (e.g., a song, a telephone number, or a dance) forwards and backwards reflects this power. The finding that motor cortices are activated in the recall of a sequence of abstract geometrical figures (Schubotz and Von Cramon, 2004) suggests that sequenced motor activity can pre-take any kind of sequenced information.

Does the brain host a “sequencer”? If so, where is this “sequencer” situated? (Buzsáki and Tingley, 2018) point to the hippocampus. The authors propose that the hippocampus produces a “sequential content-free structure” which connects to any functional area to produce a sequence of actions or sensations at recall. This hypothesis is supported by oscillatory interdependences between hippocampal rhythms and cortical rhythms in general (Feenstra and Holsheimer, 1979; Colom et al., 1988; Siapas et al., 2005). This theory however needs to be nuanced as indicated by the case of Clive Wearing (Wilson and Wearing, 1995). Wearing was a musician suffering from herpes encephalitis impairing the hippocampal area. Wearing was unable to remember anything that happened to him but could still perform as a pianist. This suggests that motor planning areas have their own “sequencers” or even that their main function is to send sequenced impulses to the body. It has been shown that the pre-motor supplementary area has “a critical role…in the organization of forthcoming movements in complex motor sequences that are rehearsed from memory and fit into a precise timing plan” (Gerloff et al., 1997).

Wearing could not remember what happened to him recently, but he could remember the past (e.g., he remembered his wife, but as he turned away from her, he forgot that she was present in the room). This is in accordance with a classical study (Scoville and Milner, 1957), stating that hippocampal function is critical to normal memory function, but not to early memories and technical skills, as well as recent findings showing that this area is critical in associative learning but not in retrieval of memories (Caviezel et al., 2020).

The Scoville and Milner study is an example of early explorations of the brain, based on conclusions from brain damage/lesion studies, “which identify *necessity*” (Dudai and Morris, 2013), leading to a segmented understanding of the brain (e.g., Brodmann's map of 52 function-specific areas). Later approaches measure *correlates* of activities leading to patterns of synchronized activations. This is developed by Anderson in the “Reuse Approach” (Anderson, 2014). The approach postulates that the same brain structure can be involved in a diversity of tasks depending on how it is connected to other brain structures. This has been compared to modern soccer (total football), where every player can take on any task (Raja and Anderson, 2019). Not even the auditory cortex is engaged solely in hearing (Anderson et al., 2013). Without the auditory cortex, we cannot hear, but hearing is just one function recruiting this area. Brain structures of are connected and reconnected depending on the task. Function depends not just on functional brain structures, but also on the structure of the pattern of structures. This is reciprocal; global structure affects the function of local structures.

The activation of reused traces depends on plasticity, which according to the Hebbian rule are synaptic connections strengthened by repeated use (Hebb, 1949). This understanding has been extended to involve oscillatory plasticity (Hoppensteadt and Izhikevich, 1996; Trevisan et al., 2005; Düzel et al., 2010; Clements-Cortes et al., 2016; Bréchet et al., 2021; Kim and Large, 2021).

Anderson's “reuse approach” provides a general explanation of how brain functions acquired “first in both evolutionary and developmental time (e.g., perception, motor control) are reused in functions that come later)” (Raja and Anderson, 2019). This suggests that the complex neural activity involved in the perception of art, can be derived from basic perception-action. Gibson argued that it does not make sense to speak of two separate kinds of perception, an ordinary and an aesthetic one (Gibson, 1975). Art is however not an ecological domain. If we are to explain aesthetic experiences by embodied theory, we must assume that faculties developed in ecology are recombined and reused in the perception of art.

Reuse in art can be exemplified with musicality. Musicality (or something like musicality) can be traced in various species such as parrots, songbirds, elephants, seals, and bats. These species can learn vocal sounds and entrain rhythms. Interestingly, they share a neurological trait with humans: the connectivity between vocal motor structures and auditory structures (Patel, 2014). This sensorimotor coupling is genetic, (Pfenning et al., 2014), reciprocal (Phillips-Silver and Trainor, 2005) and presumably oscillatory (Large et al., 2015). It is a prerequisite for imitation of sounds. Vocal imitation demands that heard sounds are linked to the production of sounds. This is how infants implicitly learn to talk and sing. It is unthinkable that a pre-lingual infant should figure out how to produce a heard word or a melody. The learning of speech and music in infants requires direct coupling. Another example: The premotor area is linked to the vestibular cortex, which could be expected since the vestibular cortex gives feedback on acceleration, retardation and change of direction (zu Eulenburg et al., 2012). It may thus not be surprising, but still is, that vestibular stimulation (by moving a listener) influences the perception of rhythm (Phillips-Silver and Trainor, 2007). Here, motor structures and the vestibular system are reused for the perception of music. It is an effect caused by a reconfiguration of the network. This demonstrates that the pre-take of rhythm cannot be attributed to one brain area but to a pattern of reused functions. This pattern enables us to connect in collective rhythmic coordination. It may take various expressions such as rituals, dancing, playing, singing, hand clapping, marching to military music, working to works-songs or just waving a lighter at a concert. This coordination does not need a specific goal. However, it fulfills a universal need to connect with other people. The theme of synchronization and socialization will be developed further in the paragraph on empathy.

These examples of are just two out of a range of connections in a network activated by music. Endogenous network activity provides the context to perceive music as music rather than as unorganized sounds. Music is a play with faculties developed in ecology. In this respect, it exemplifies the Reuse Approach.

The Reuse Approach is an embodied approach in that it states that cognition cannot be separated from bodily activity “…action does not come after thinking, which comes after perceiving; thinking, perceiving, and acting are synchronous and co-determining” (Anderson, 2016).

To conclude:

1. As argued with the example of Pavlov's experiment, associative learning embodies declarative knowledge. All knowledge is embodied irrespective of if it concerns skills or symbolic knowledge.
2. Associative memory concerns the association of two elements. It could be considered the building block of sequences. This provides information of *what* will happen next in a chain of events. *When* (the timing of this event) is decided by motor planning activity providing an endogenous temporal chain of information. As can be concluded from the research of music, endogenous oscillatory activity provides an exact schema of pre-takes. Is this general? Does it apply to the perception of static art forms, such as paintings and sculptures? We will come back to this question.
3. The species-specific structure of the nervous system (DNA information), individually modulated by plasticity (perceptual leaning), pre-takes the work in a network of reused traces.

Contemporary enactive embodied theory implies a reorientation of traditional understanding of the brain. The pioneers in neuroscience investigated consequences of brain damage, leading to a segmentation of the brain into Brodmann's areas connected by neural pathways. The use of new techniques allowing for studies of the electrical activity in the brain provides evidence inspiring theoretical development. The trend in this development is the shift of research interest of areas communicating via neural pathways to synchronized patterns.

For the understanding of “context” in perception, this implies a reactivation of traces in a neural network paved by previous perceptions. Since these traces always entail motor activity and specifically motor planning activity, context is sequential. It is pre-taking.

This is the cognitive side of perception. Taking into account that cognition and emotion are reciprocally dependent, the inner state of the perceiver is an interaction of associations (activity in reused traces in the manifold of possible connections paved by plasticity) and emotion. This makes up the internal state of the perceiver and this whole state is contextual in any perception, including the perception of artistic artifacts or performances.

## The state of the artist

“Aesthetics is for artists as ornithology is for the birds.” Barnett Newman, American artist and founder of ‘abstract expressionism, quoted by (Levinson, 2007).

If aesthetics has no relevance for the artist, what then is the perspective of the artist at the state of creation? The Swedish conductor Gunnar Eriksson, looking back on his life in music, claimed that artists continue to play for the rest of their lives.^1^ Make-believe games are acts of imagination. Art is a game, although sometimes a serious one.

“Imagination is a guide to possibility” (Kind, 2005). It requires an actualization of knowledge—knowledge in the sense of “what” (the toys in the play) and the sense of “how” (the toys move and interact). Imagination is a mental simulation of a sequence of events. A memory called to mind is an imagination, but imaginations can be open ended. They are not always faithful to earlier experiences. They allow for new combinations in the play.

In a sequence, the next event is suggested by the sequence so far (as “pre-takes”). A sequence of neural events can be perceived—envisioned, sensed or heard. This is called *imagery*—sensations, which do not have an external source. Imagery is a central aspect of imagination (McGinn and Mac Ginn, 2004). Since the word “imagination” means visualization, the concept implies that something is more or less obviously played up to the senses. Imagination and imagery are grades on the same scale.

Imagery is an important faculty in creativity. Taking a walk, you may start thinking about a recent situation, where you were insulted. You start arguing in your mind. You can hear the opponent talk back, and now you are involved in a dialogue. You act out the situation. Your lips may move. You may be gesturing. You perceive the exact sequence of words (and syllables). It is a chain of pre-takes. It is an “as-if” game. It is also an embryo to a scene in a play if you are a playwright. For a composer experiencing *musical imagery*, it just remains to put it down, record it, or simply play it. For the improvising musician, music imagery goes right into the instrument. It is already connected to motor sequences.

Musical imagery can be heard as anything from looped sequences to fully orchestrated music, perceived completely in all properties (Halpern, 2012). This elucidates how Beethoven could continue to compose, in spite of his loss of hearing. The research of the phenomenon provides insights concerning contextual embodied knowledge and perception. Musical imagery has been connected to activity in a network overlapping with the network for music perception, with the exception of the primary auditory cortex (Kraemer et al., 2005). It does however activate the auditory associative cortex.

Musical imagery is predictive (Zatorre et al., 1996). We can hear a continuance of interrupted music and even the beginning of the next song on a CD. Since this prediction does not have an external source, it is purely endogenous. Since it is predictive, it is contextual.

Amazingly, neural oscillations resonate not only with musical rhythms as demonstrated by the research of Large and associates but also with imagined rhythmic properties (Zoefel et al., 2018). Examples of imagined rhythms are the prediction of the next beat in music and the almost inevitable feel of meter (1/2; 3/4; 4/4; 6/8 etc.) in isochronous pulses such as the ticking of a clock, or our walking steps. Zoefel's finding demonstrates that imagination is directly connected to endogenous activity. Endogenous oscillations are also involved in the retrieval of learnt motor sequences (Pollok et al., 2015).

A role of endogenous oscillations was suggested by (Jones and Boltz, 1989), claiming that synchronization of endogenous and exogenous oscillations generate expectancies for future events, where endogenous oscillations designate brain oscillations and exogenous oscillations designate the musical soundwave. A row of studies confirm that such expectancies “enable attentional coordination with the environment” (Large et al., 2015). This is to say that the musician and the listener are coupled in a mutual pre-take. “Attentional coordination” is to experience music when played, in spite of neural delays.

The research of musical imagery demonstrates:

1. That, when the external music stops, the exogenously induced activity ends, but the predictive endogenous activity goes on and can be heard as imagery.
2. That, listeners have implicit knowledge concerning organizational principles of musical styles. Otherwise, the nervous system would not be able to produce music imagery. This means that we have *implicit models of music* creating “weak anticipations”. These models are not stored as books on a shelf, but simulations of previous perceptions enabled by plasticity.
3. That, we can retrieve a load of tunes, replayed by the brain, and heard as imagery. These are thus, in this sense, *re-presentations*.
4. That, these “re-presentations” can be mental, since they can be heard (as music imagery).
5. That, these “re-presentations” are neural, since music imagery has no other source.
6. That, “attentional co-ordination” entails synchronization with the environment in perception-action. Endogenous oscillations and exogenous oscillations are synchronized. This is in accordance with ERP-research, with Dubois' understanding of synchronization as a pre-take, and with Neural Resonance Theory and research.

Musical imagery is a mental representation, since it is heard. This is not to say that mental representations are involved in perception, but that it is evidence of endogenous activity providing information for a pre-take.

This understanding deviates from “radical embodied cognitive science”. Information may be picked up by the nervous system, much as the strings in a piano resonate to a voice singing into it. We must however take into account that this piano is also self-playing.

The mechanical activity of a self-playing piano is not music, but it is perceived as music. By the same token, endogenous brain activity is not music, but it can be perceived as music. This requires that endogenous activity “plays” on the sensory field. It has recently been demonstrated that the anticipation of a sound is a sensorimotor driven activation of auditory cortex (Gelding et al., 2019). Sensory field activation has also been reported for visual imagination (Slotnick et al., 2005). Wheeler et al., 2000 found that retrieving vivid visual and auditory information reactivates some of the same sensory regions initially activated in its perception. This has also been observed for spatial information (Persson and Nyberg, 2000). These studies suggest that the description of how neural activity can “play” on the sensory field and produce imaginations in the case of music, is general. It is not restricted to music.

All and all, the research of music demonstrates:

1. That, musical imagery is endogenous sequenced activity presenting pre-takes to the sensory field.
2. That, the brain can simulate music.
3. That, this simulation can be heard.
4. That the pre-take is mandatory in composing and playing. The continuance of the piece is continuously suggested to the composer as the sequence unfolds.
5. That the pre-take of the composer is soundwave of the composition.
6. That endogenous oscillations provide information without which music cannot be perceived as music. In this understanding, musicality is the ability to pre-take the next event in the flow of music.

There are two “streams” in the nervous system in the listener. One “stream” of sequenced oscillatory neural activity induced by exogenous soundwaves and another “stream” of sequenced endogenous oscillations, where the endogenous “stream” is a pre-take and thus contextual. Importantly, these streams are not streams along neural “pathways” from one brain area to another, but “streams” in the sense of sequenced oscillatory activity over time.

To conclude:

Since the music is a manifestation of a row of pre-takes, the beholder perceives the pre-takes of the artist. Since perception depends on pre-takes, the beholder pre-takes the pre-takes of the artist. Viewed as an information system encompassing the artist, the work of art and the beholder, this means that shared context leads to inter-brain synchronization. Pre-taken information is omnipresent in the system.

This implies:

1. That, the neurophysiological state of the artist at the point of creation is contingent of synchronous and co-determining activity in a network consisting of traces left by perceiving, modulated by, but also producing, emotions.
2. That, similarly, the neurophysiological state of the beholder at the point of perception is contingent of synchronous and co-determining activity in a network consisting of traces left by perceiving, modulated by, but also producing, emotions.
3. That the mental state in both cases corresponds to endogenous neural simulations “playing” on the sensory fields producing imaginations.
4. That the state in both cases is continuously changing.
5. That the correspondence of these imaginations depend on interbrain synchronization of endogenous simulations.

This paves the way for an understanding of art in terms of empathy—as shared internal states.

## Aesthetic empathy

“Art is not a handicraft, it is a transmission of the feeling the artist has experienced” (Tolstoy, 1995). This formulation captures an understanding of art in just one sentence. It connects art to empathy.

Empathy has been defined as “…our ability to perceive both *that* as well as *what* another is thinking and feeling and to develop a felt response to these perceived thoughts and feelings” (Krueger, 2009). This definition is one of many, but it may serve as a starting point.

Empathy applied to art can be exemplified:

1. Empathy with the creator. We can feel the mood of a composer (e.g., Gustav Mahler's melancholic maelstrom in the Fifth Symphony).
2. Empathy with a performing artist: Watching a tightrope dancer, the German philosopher Theodor Lipps exclaimed: “*…Ich Fühle mich so in ihm*”(I feel myself in him) (Lipps, 1903). This can also be exemplified with audience reactions on Mick Jagger acting out “Satisfaction”. “Empathy” is in fact an understatement of the explosion of sexual love and identification with raw models in teenage mass audiences at rock-concerts in the 60s. The spectators were sometimes so overwhelmed my compassion that they passed out.
3. Empathy with a depicted person, as expressed by the 15th century Italian architect Leon Alberti: “The painting will move the soul of the beholder when the people painted there each clearly shows the movement of his own soul...we weep with the weeping, laugh with the laughing, and grieve with the grieving. These movements of the soul are known from the movements of the body” (Alberti, 2005).

Empathy is a contested concept. We are faced with forty-three definitions (Cuff et al., 2016). The word “empathy” is a Latin-Anglicization of the German “Einfühlung”—the ability to feel in. “Einfühlung” was used by German philosophers in the late 19th century in the discussion of aesthetic experiences of artworks. Originally, this concerned how we project ourselves into the artwork and only later, the ability to feel into another person. Here, these two understandings will be combined.

I will present an enactive embodied understanding of empathy, entailing Anderson's “reuse” concept, Dubois” “strong and weak anticipation” concept and lead this to Chalmer's and Clark's “extended mind theory”. As a point of departure, let us go to Edith Stein's phenomenological understanding of “empathy”.

Edith Stein, a student of Edmund Husserl (the founder of phenomenology), regarded empathy as a *sharing of mental states*. In her pioneering seminal thesis *On the Problem of Empathy* (Stein, 1917), she draws on the concept of intersubjectivity, introduced by Husserl as “more than shared or mutual understanding and closer to the notion of the possibility of being in the place where the other is” (Duranti, 2010). This is perspective taking. The concepts of “shared mental states”, “intersubjectivity” and “perspective taking” all reflect shared context.

The contemporary discourse on empathy involves numerous sub-concepts, summed up by (Batson, 2009, p. 3). This has led to a “multicomponential construct”, inspired by Scherer's model of appraisal (Scherer, 2001). Appraisal theory claims that emotions are deduced from evaluations (appraisals or estimates) of events. This theory thus implies that emotions depend on inferences. Appraisal is often divided into several subcomponents such as conclusions from cues, intentional perspective taking and situation evaluation. This makes the theory of empathy akin to the *Theory of Mind* (Woodruff and Premack, 1978). Woodruff and Premack argued that empathy must be inferred from cues, since we do not have direct access to the mind of the other.

The meaning of empathy thus has changed from Stein's phenomenological *experience* of the mind of the other to multifaceted umbrella concepts involving conclusions resulting in an *understanding* of the mind of the other, leading to emotional reactions. Appraisal theory is a commonly accepted approach to emotion in psychological literature. This is reflected in articles, aiming to encircle what an aesthetic emotion is. For example, Menninghaus states that aesthetic emotions “always include an aesthetic evaluation/appreciation of the objects or events under consideration” (Menninghaus et al., 2019).

The general problem with the appraisal theory of emotion is not so much the involvement of cognition as the assumption that emotions are caused by a cognitive evaluation of a situation and that emotions are inferred from cues. Shusterman reverses this assumption: “Evaluative outcomes are evoked through the emotions associated with the bodily and behavioral changes that occur during an interaction” (Shusterman, 2013).

In the case of music, it has been shown that “…music may recruit neural mechanisms similar to those previously associated with pleasant/unpleasant emotional states, but different from those underlying other components of music perception” (Blood et al., 1999). Peretz research on “amusics” shows that they have normal emotional reactions to music although they do not have conscious access to musical parameters (Peretz et al., 1998; Peretz, 2016). Both studies indicate that experienced emotions (feelings) in music listeners do not depend on conclusions from what we hear. As we have seen, there is a preconscious ERP-reaction, which precedes awareness.

What about literature? It would seem that this art form engages cognitive functions and that the emotions of the reader depend on the understanding of the fictional situation. Undoubtedly, but even this understanding is embodied. Reading ability depends on associative learning as argued above, where the symbol is associated to perception-action experiences.

The body has a “meaning generating role” (Colombetti, 2010). Colombetti states that this role has largely been disregarded by emotional science, which “tends to attribute this role only to separate abstract cognitive-evaluative processes”. Such models, she says, “postulate a Cartesian brain operating on its own, distinct from the body” (i.e., computational theory). Colombetti refers to Antonio Damasio, who presented an understanding of emotion as “…an organismic process of self-regulation aimed at maintaining homeostasis. Emotion thus conceived also provides action-guiding values, drives and preferences” (Damasio, 1999). Colombetti concludes that sense making involves emotion. Embodied theory, she states, “is as much a theory of emotion as it is a theory of cognition”. On these grounds, Colombetti is critical of the appraisal theory of emotion.

The view that we can share mental states, without reasoning, is gaining territory through a growing, widely branched body of research. This research is interrelated and the hypotheses and models are partly overlapping. It points to an automatic reciprocal connection between motor activity and emotion. This view does not deny cues, but concerns how cues are acted on (i.e., pre-consciously).

This research comprises:

1. *The facial feedback hypothesis* stating that our own facial expressions affects our emotions. This idea can be traced back to Charles Darwin's *The Expression of the Emotions in Man and Animals first* published in 1872 (Darwin and Prodger, 1998). The hypothesis has been widely tested. A recent meta-analysis of 138 studies confirms small but significant effects (Coles et al., 2019).
2. *Emotional contagion*—an idea developed by Elaine Hatfield. It concerns the tendency to automatically mimic “movements, expressions, postures and vocalizations with those of another person and consequently converge emotionally” (Hatfield et al., 1993). The research of mimicry demonstrates that we automatically mimic the expression of conspecifics and that this comprises motor as well as autonomic/physiological expressions (blushing, skin conductance, heart rate variability etc.) leading to a tendency of synchronization between interacting subjects. Connected to the facial feedback hypothesis, this leads to empathy through mimicry (Prochazkova and Kret, 2017).
3. *Rodent empathy* research demonstrating that that “rodents are capable of empathy, a process where the affective feelings of one are conveyed to another and then generate the same feelings in that individual” (Panksepp and Lahvis, 2011). Rodent empathy exemplifies that empathy does not require understanding, unless we have seriously underestimated the cognitive abilities of mice.
4. *The perception-action model of empathy*, which states that the perception of an emotional expression activates brain areas required to produce the same expression, which in turn elicits the adequate emotion in the beholder (Preston and De Waal, 2002). This model is inspired by the discovery of the mirror neuron system (Di Pellegrino et al., 1992).
5. *Mirror touch synesthesia. “*Watching another person being touched activates a similar neural circuit to actual touch and, for some people with “mirror- touch” synesthesia, can produce a felt tactile sensation on their own body” (Banissy and Ward, 2007). The authors show that the phenomenon correlates with heightened empathic ability and conclude that we “empathize with others through a process of simulation”.

This research is commensurable with three crucial points, presented above:

1. The observation of an activity, activates the same sensorimotor network as when we perform the activity ourselves
2. This activity is pre-taken.
3. Motor activity is correlated with emotion.

Contemporary enactive embodied science displays a return to the original phenomenological understanding of empathy. This can be exemplified with Krueger's “extended, bodily skills–based view of empathy and intersubjectivity” drawing on *the* “extended mind thesis” (Krueger, 2009). This thesis, proposed by Clark and Chalmers, claims that cognition is extended outside the nervous system (Clark and Chalmers, 1998). Where information is picked up in the environment, the scull is not the limit. This idea connects with concepts of embodied theory such as Merleau-Ponty's *body-schema* (Merleau-Ponty, 1945) and Gibson's concept of *affordance*, since both include environmental features (instruments, which through habitual use become incorporated in the body schema, and action possibilities provided by the environment, respectively). Clark and Chalmers specify: “Mental states are (potentially) composed of neural, bodily, and, most controversially, worldly, properties—such as tools, artifacts, and technologies; language and other symbolic representations; environmental affordances; sociocultural institutions; and other minds” (Clark and Chalmers, 1998). As can be noted from this quote, artifacts (e.g., a work of art) and other minds (e.g., the artist) are components of the extended mind of the beholder. The extended mind theory does not dissolve the organism into the environment, since a living system is an *autopoietic* (Greek: self-created) and self-maintaining system, which defines its boundaries (F. G. Varela et al., 1974). Rather, this self-maintaining system is part of a larger cognitive system.

The extended mind is often exemplified with the cell phone. The phone performs cognitive functions such as navigation, memory, communication, and calculation. Importantly, after some time, the user interacts with it automatically and directly. The thumb operating the phone has a life of its own. The knowledge of how to use the tool becomes embodied. If so, it does not matter whether the phone is inside or outside the scull. The same can be said about the tools in art. The brush of a painter, the guitar of a flamenco artist, the computer of an author serves after habituation as an extended part of the body and thus of cognition. They are parts of the *body schema*. Then, painting is just like visualizing; playing an instrument is just like singing; and typing is just like talking. As mentioned, imagery is an important capacity in art production. It engages sensorimotor functions. In the case of music, the sheet is often a transcription of musical imagery (i.e., the composer writes down internally heard music). The score contains information for the musician regarding the endogenous sequence in the composer. For the experienced musician, the score is sounding with music. This is a variant of music imagery. Just as the composer can hear her own endogenous brain activity, the musician can hear the endogenous activity of the composer by a look at the sheet. The music coming out of the orchestra is in this sense literally the extended mind of the composer in the act of creation. The orchestra sounds with sequenced endogenous activity. In the end, this is what the listener perceives (provided that the music is not calculated, as is often the case with atonal music). In most cases, composing involves imagination as well as calculation.

Extended cognition raises the question:

*Can a work of art simultaneously be “extended cognition” of the artist and the beholder?*

Followed by the question:

*Is “extended cognition” the key to aesthetic empathy?*

If so, art can be analyzed as an “extended cognitive system” encompassing the artist, the work, and the beholder. This gives the beholder access not just to the work, but to the mind of the artist, which is the ultimate understanding of the extended mind.

The Austrian phenomenologist Alfred Schütz farsightedly expressed this part taking in an essay contemplating music listening in the 50's:

“Although separated by hundreds of years, [the listener] participates with quasi-simultaneity in [the composer's] stream of consciousness, by performing with him step by step the ongoing articulation of his musical thought. The beholder, thus, is united with the composer by a time dimension common to both.” (Schütz, 1951).

Here, Schütz describes a mental synchronized *co-performance* of the artwork. He emphasized the shared time resulting in a unification with the artist. He calls it “quasi-simultaneity”. It is a synchronization with an event hundreds of years ago, bridged by the note system or recording technology. The beholders of temporal art forms enact a sensorimotor script. This makes temporal art forms ritualistic (Vickhoff, 2020). This theme will be developed in the discussion.

Patterns of synchronization have been found from the simplest animal life forms to humans (Couzin, 2018). This omnipresence of synchronization supports Neural Resonance Theory (i.e., the nervous system obeys general physical laws of resonance). Synchronous activity has an impact on empathy assessed as prosocial behavior. In humans, studies demonstrate that synchronized movement positively affects affiliation (Kokal et al., 2011) as well as cooperation (Trainor and Cirelli, 2015). In a recent article called “Higher empathy is associated with stronger social bonding when moving together with music” (Stupacher et al., 2021), the authors accounts for a series of experiments enlightening the claim in the title. In connection with this, it is interesting to note that the administration of oxytocin—a peptide associated with empathic behavior, improves finger-tapping synchronization between a leader and a follower (Gebauer et al., 2016). Thus, the ability to synchronize seems to be connected to social interest. This can be associated with bird courtship, where the male and the female of some species engage in synchronized rituals, and even with human courtship, where the dance floor provides a scene for inter-personal synchronization. On a grand scale, as in the case of electronic music at rave parties, the whole floor resonates with the rhythm, resolving the limits between individual dancers. It is not you and me; it is *We*. It is an extension of cognition—a resonating system.

A recent article on empathy showed that…

“…sharing a painful experience triggers emotional resonance between pairs of individuals through brain-to-brain synchronization of neuronal α-oscillations recorded over the sensorimotor cortex, and this emotional resonance further strengthens social bonds and motivates prosocial behavior within pairs of individuals” (Peng et al., 2021).

Interbrain synchronization requires parallel endogenous activity. Endogenous activation corresponds to imagination in the artist and to context in the beholder. As argued in the preceding paragraphs, context can be non-representative, representative, preconscious or conscious.

This suggest three levels of aesthetic empathy:

1. Pre-conscious non-representative endogenous activity (i.e., strong anticipation) matching exogenously induced activity (by sensory input from the work of art).
2. Pre-conscious representative endogenous activity (i.e., weak anticipation based on models) matching exogenously induced activity.
3. Awareness. The beholder experiences like-mindedness between herself and the artist.

It should be noted that it is just the third stage that approximately fits contemporary psychological definitions of empathy accounted for above. The first two are prerequisites to the third stage. All three involve emotion. In order to have an existential dimension (i.e., to mean something to our lives), art must evoke autobiographical memories providing a sense of who we are. As mentioned, the fact that the motor activity correlates with memories (Ianì, 2019), indicates that the motor sequence activates a network of reused traces. In behavioral terms, the sensorimotor sequence is a mnemonic to a variety of memories.

This entails three possible scenarios:

1. A mismatch of endogenous and exogenous information. This is the *prediction error*, requiring a reset of the endogenous sensorimotor sequence, which, as argued, actualizes new context.
2. If the artist and the beholder have the same sensorimotor information, the artwork is enacted. Brain functions evoked by the work of art result in synchronized exogenously induced activity and endogenous activity in the beholder, entailing synchronization between the artist and the beholder.
3. Sensorimotor information is shared, but autobiographical memories linked to sensorimotor activity are individual, since we follow different trajectories in life. We may however share autobiographical memories to a certain extent through similar cultural background.

To conclude:

Aesthetic empathy in terms of enactive embodied theory implies that the artist and the beholder enact the work of art and thus each other. They *pre-take* each other and *co-perform* the work. This adds a dynamical aspect to the understanding of empathy. Empathy is not just a shared internal state; it is a synchronization of a sequence of states. Empathy can be preconscious or conscious. If the artist and the beholder share *art-context* (e.g., the embodied knowledge of the style), we have a case of synchronized enactment of the work of art. The co-performance of sensorimotor sequences links to *non-art context* (e.g., autobiographical memories), which may be overlapping but not identical. There can never be complete aesthetic empathy. The non-overlapping space is Danto's *ellipsis*—the freedom of interpretation.

This understanding of aesthetic empathy corresponds to “extended mind theory”. The cognitive system comprises the beholder, the artifact (the work of art), as well as the artist. Information is shared and synchronized in the system.

Next, I will apply this understanding in a conceptual framework of musical empathy. Generalizations to aesthetical empathy will be addressed.

## A conceptual framework of musical empathy (and beyond…)

**Figure 1:**
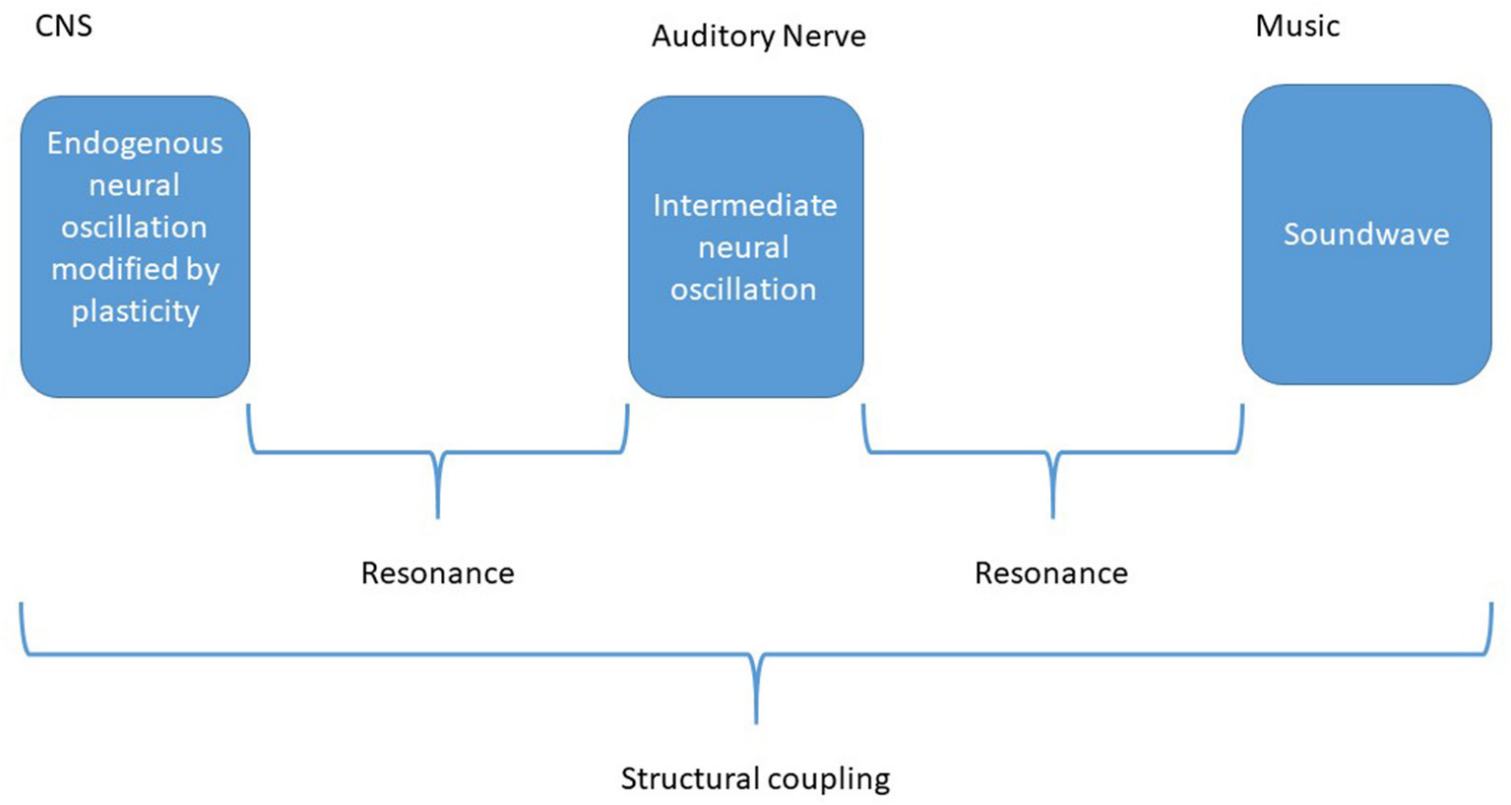
Structural coupling in music perception. The listener is structurally coupled to music *via* two steps of resonance. The structure of the soundwave is converted to electrical potentials in the cochlea and picked up by the auditory nerve by way of neural resonance. The structure of endogenous oscillations is immanent in reused motor-planning (sequenced) oscillatory traces. At music perception endogenous oscillations pre-take auditory nerve oscillation.

**Figure 2:**
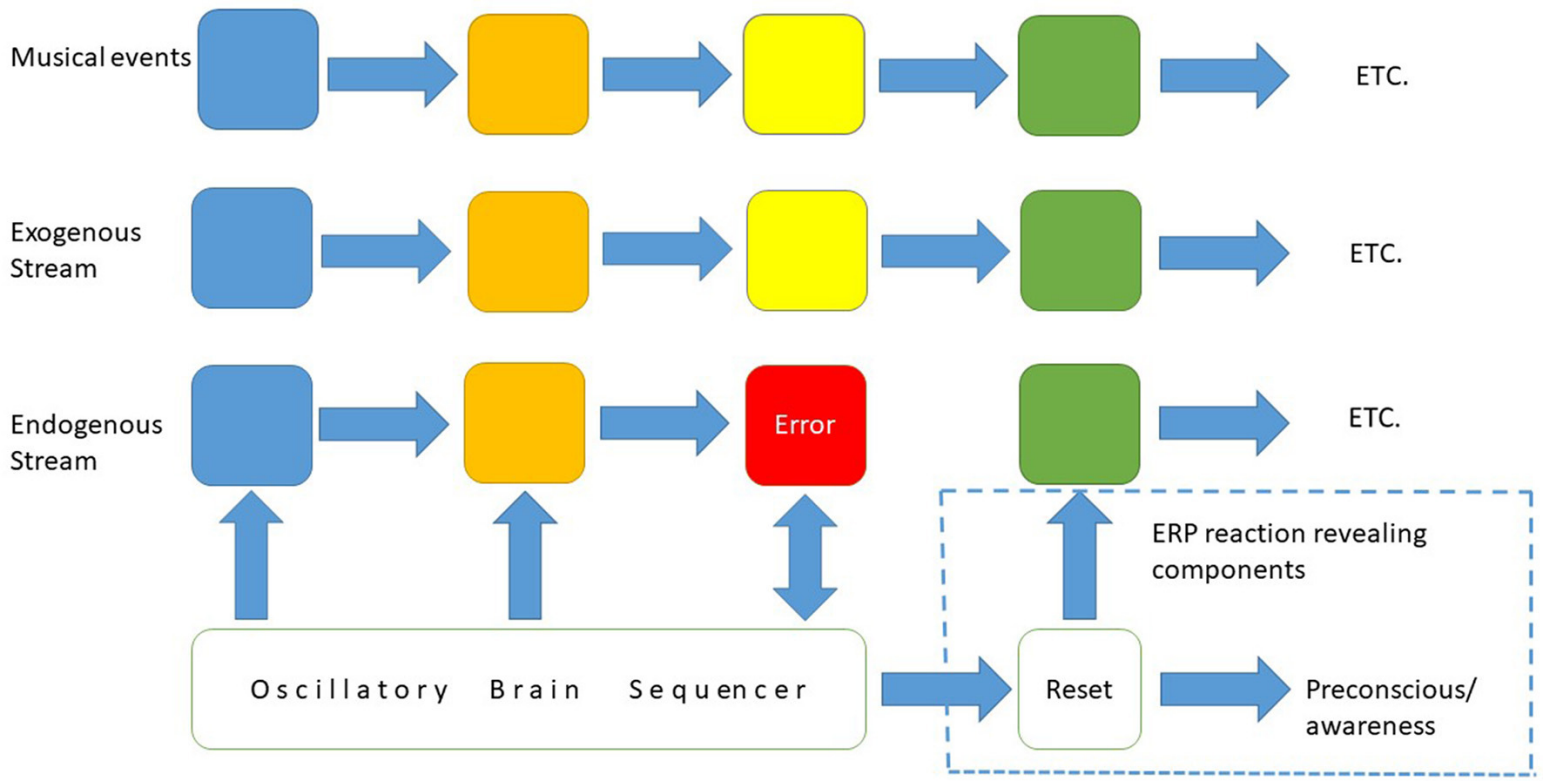
Musical enactment/mismatch. The figure shows interaction between the exogenously induced sequence (in the auditory nerve) and endogenous sequences pre-taking oscillations (presented in Figure 1). Matches in color signify structural coupling (matching information). The event (in red) is a mismatch (a desynchronization of sequences), detectable in ERP as the “prediction error”. This implies a reset of the endogenous pre-taking sequence. ERP reaction times separate components in ERP potentials: reactions on strong anticipations from weak anticipations, and unattended mismatches from attended mismatches.

**Figure 3:**
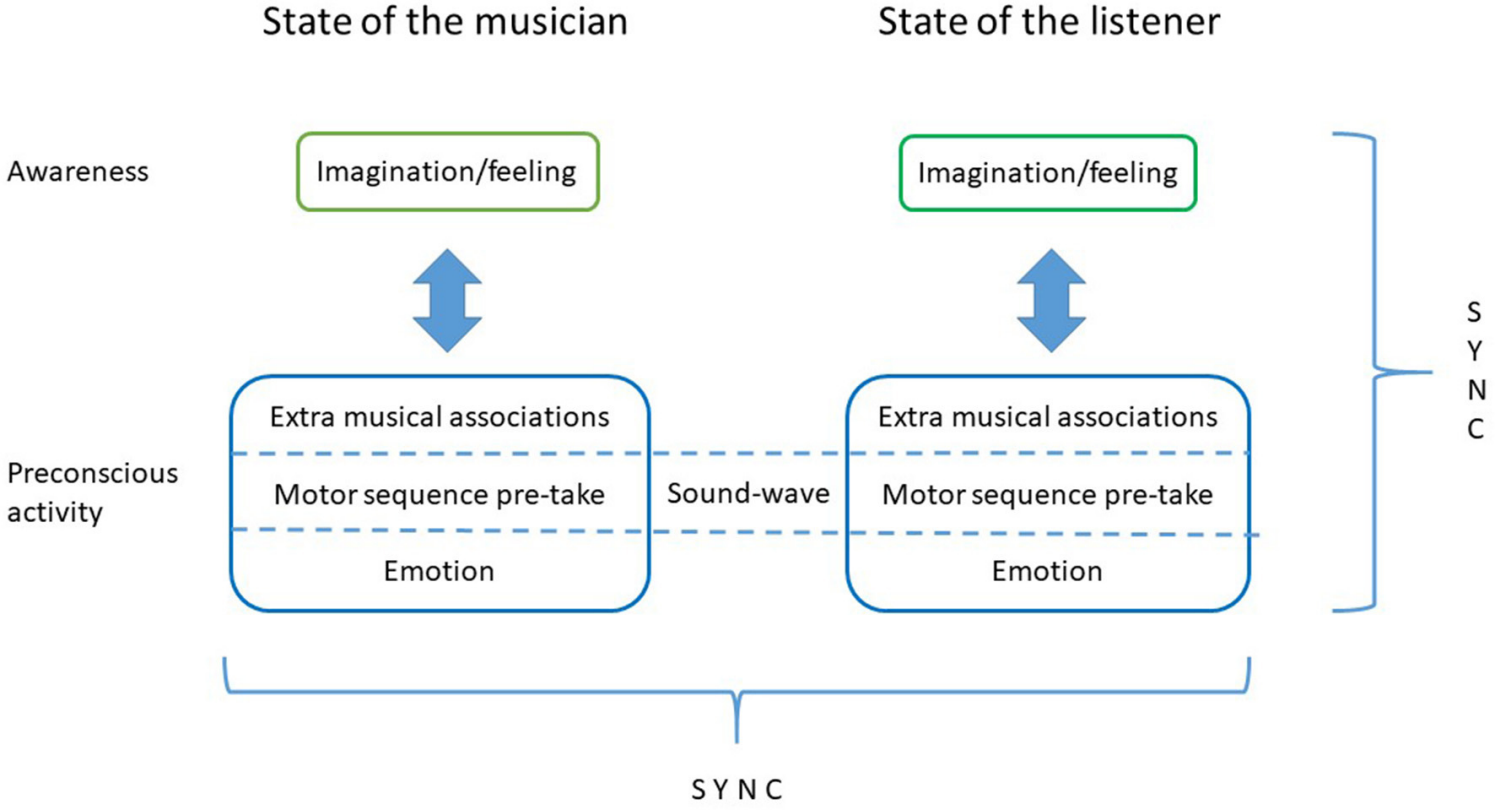
Musical empathy in an extended cognitive system. The musician and the listener both pre-take the music as it unfolds. It is a mutual enactment. Imagination is awareness of endogenously pre-taken music. The artist performs her neural simulation. The listener endogenously co-performs the work. The artist and the beholder are coupled. The motor sequence is associated with extra-musical memories as well as with emotion.

This framework is based on the findings presented to far. Since the evidence is biased toward the neuroscience of music, I will exemplify aesthetic empathy with the case of music. This will be followed of by a discussion of appliance to other art forms. The framework for musical empathy is introduced in three consecutive figures. Figure 1 shows structural coupling. Figure 2 shows coupling/mismatch in ERP-research. This leads to Figure 3, showing musical empathy as a system including the artist, the beholder and the music. Interbrain activity comprises cortical as well as subcortical activity correlating over time between music listeners resulting in consistent and reliable patterns of inter-individual time-locked brain activity (Abrams et al., 2013). The framework suggests that intra-synchronized networks are inter-synchronized at empathy. The concept of synchronization is used in a general sense (Pikovsky et al., 2001).

Resonance implies reciprocal modification of oscillatory activity. The oscillations in the auditory system are thus modified by endogenous oscillations. In behavioral terms, this is to say that anticipation modifies perception. On the other hand, exogenous oscillations modify endogenous oscillations. Resonance implies synchronization. The music is co-performed by the listener as a neural simulation. The music is enacted.

Structural coupling is indirectly demonstrated in ERP-research, since ERPs are reactions of the opposite (i.e. mismatches). This is shown in Figure 2. As long as there are no mismatches, the information is synchronized. At mismatches, the brain reacts. The prediction error reaction indicates desynchronization of the endogenous sequence and the exogenously induced sequence. The mismatch leads to a reset of the endogenous sequence—a re-synchronization. Analysis of ERP reaction times separates components of the potential as mismatches of “strong anticipations” (pure mismatch of information) from mismatches of “weak anticipations” (based on models). In addition, it separates preconscious from conscious reactions (experienced surprises).

The enactment in Figure 2 connects the beholder to music. The interesting event is the prediction error. The desynchronization of the sequenced variables triggers a reset of endogenous oscillation, which corresponds to new context. This context does not just concern the music, but also a new set of associated memories (e.g., the name of the performer, earlier encounters with the music etc.).

There are three couplings in music perception:

A. Synchronization between sequenced musical events and the Auditory Nerve sequence.
B. Intra-brain synchronization between the Auditory Nerve sequence and the sequence in reused traces.
C. Intra-brain synchronization binding reused traces to a synchronized network binding functional areas to coherent perceptions and memories.

These couplings lead to synchronization between the musician and the listener. The Auditory Nerve sequence is largely the same in both agents since they receive the same input from the music and have the same species-specific hearing structure. If the musician and the beholder have the same history of musical experiences, they have the same sequential pre-taking of musical events, leading to implicit or explicit synchronized co-performance of the music. This synchronization is a prerequisite for our ability to play together (Vickhoff, 2019). Figure 3 demonstrates how the preceding pictures lead to a synchronized, reciprocal and symmetric cognitive system—an extended cognitive system.

In the artist, a possibility of combined reused traces is presented to the mind as imagination/imagery. This is the vision she performs. It is already coupled to motor activity. The performance is a continuous inner “dialogue” between endogenously driven imagination and exogenous feedback from the work in progress, resulting in confirmations or prediction errors depending on her *skill* (precision in sensorimotor couplings).

In the beholder, the exogenous stream consists of the sensorimotor information in the work. For perception, this sequence must be related to context—the endogenous stream, consisting of congenital and reused endogenous activity.

Since the input from the work is virually the same for the artist and the beholder, similarities and differences in perception depend on similarities and differences in context. If exogenous and endogenous motor sequences match and correlate, the work is *co-performed* as a performance of the artist and as a simulation in the beholder. Correlated exogenous and endogenous sensorimotor sequences open a wider span of endogenous reused activities. In behavioral terms: correlated motor activity is a mnemonic to memories, providing an experience. Musical empathy is extended cognition, where the extended mind is just the surface.

Which are the prerequisites for generalizing this framework of musical empathy to other art forms? Music is special in the pronounced synchronizing effect of rhythm. “…the auditory system is better suited to guide temporally precise behaviors like sensorimotor synchronization (SMS) than the visual system” (Comstock et al., 2018).

It should however be noticed that…

1. Enactment is a general consequence of perception-action, since perception-action is a basic unit in perception. This entails that perception always has a motor ingredient. So has contextual information (since it is re-activated perception).
2. Associative memory enables embodiment of symbolic presentations such as literature.
3. Strong and weak anticipation theory is generally applicable to biological systems.
4. ERPs are not limited to auditory violations; it has also been assessed for visual mismatches (Bernat et al., 2001).
5. The function of endogenous oscillatory activity is not limited to the perception of sound. Neural oscillations are also involved in visual, olfactory, motor perception systems (Bahmer and Gupta, 2018). For the visual system, it has been suggested that temporal coupling of neural activities is involved in the perception of size and continuity of spatial features (Neuenschwander and Singer, 1996). It has recently been proved for small animals that “visual selection is achieved by phase-controlled endogenous 20 to 30 Hz oscillations that lock onto temporal features of attended visual objects” (Grabowska et al., 2020). Concerning the motor system, it has been demonstrated that firing rates in medial prefrontal cortex correlates to hand and eye movements (Wang et al., 2018).

For these reasons, generalization to other temporal art forms is possible. Considering static art forms generalization is more problematic. It has been argued that is possible to feel the rhythm in paintings (Freedberg and Gallese, 2007). The authors exemplify with the works of Jackson Pollock, through simulation of sensed movements of the painter. For a nuanced understanding of this claim, see oppositional articles of Gaiger and Durà-Vilà (Gaiger, 2018; Durà-Vilà, 2019). In representational art, we do not need to go to such lengths. If, for example, a *stilleben* (still life) contains a glass of wine, we perceive that glass by earlier perception-action encounters with glasses. (grabbing them, drinking from them, the feel of holding them to our lips, and so forth). This actualizes the whole situation: the taste of wine, the color, the social experience, the emotion etc. Just as the tea-soaked madeleine cake in Proust's *Les Temps Perdu*, it re-presents the past. This concerns bodily activity required to perceive as well as bodily reactions coupled to emotions. In addition, emotional pictures affect the motor evoked potential, as mentioned above. It is also arguable that the beholder perceives the position of a depicted being as a section of a sequenced movement. If the motor sequence is known, we can feel the movement even if the depicted object does not move. If we, for example, know how to throw a discus, we may feel the movement in the *Discobolus of Myron*—the antique sculpture of a discus thrower.

There is however a more direct answer to the question of generality:

*Perception is temporal, irrespective of if the work of art is temporal or not*.

Perception-action implies that perception always involves motor activity. Motor activity is sequenced over time. It follows that the *perception of all art forms is sequenced*. Viewing a painting or a sculpture, is to scan it with our eyes. This is to sequence the perception, allowing for pre-takes. We can pre-take structures and colors in a painting as our gaze moves over the canvas, if we have learnt a model of the style. Static art forms unfold over time in the act of perception-action. The detection of an unstylistic element in a painting is an “event” (a mismatch). It is violating the pre-take. Would an unstylistic element in a painting be detectable as an ERP-reaction? So far, the design of ERP experiments on visual art perception involves presentation of a sequence of pictures (Else et al., 2015) with presentation times around one second for each picture. This is not ecologically valid. It is not how we perceive paintings. Rather, we contemplate the work as our gaze wanders over the painting.

It should however be noted that motor activity associated to scanning a work of art is not a co-performance of the work. This is an important difference between static and temporal art forms. This difference is critical for empathy, with negative implications for the therapeutic value of static art forms. These aspects will be discussed in the following.

## Discussion

Why art? The main problem in The field of Art and Health is to state the role of the arts. The field is in need of a theory providing a definition of art in order to validate what is and what is not an art effect. The step taken here is to connect “context” in the philosophical concept “aesthetic contextualism” with embodied knowledge. In this conclusive discussion, I will connect the framework to psychosomatic health and suggest operationalization of key concepts.

In the presented framework, art is considered as the dynamics in a system involving the artist, the work of art and the beholder. In this system, art is a skilled manifestation of imagination, where imagination is a hypothetical possibility proposed to the senses by endogenous activity. The expression of imagination in a work of art demands skill. Skill does not just concern the production of art works; it also concerns the perception of art works. We need to know how to do something to imagine hypothetical actions. We need to know how to do something to recall ourselves doing it. We need to know how to do something to perceive the doings of others. The artist imagines. So does the beholder. The dynamics in the system depends on the correspondence of the internal states of the artist and the beholder, where states are endogenous activities corresponding to cognitive/emotional activity. This is analyzed in terms of empathy. Art, discussed at this level, concerns the synchronization/desynchronization of sequenced interbrain endogenous activity.

Desynchronization implies conflicting information—information, which does not fit into context (such as news and surprises). At complete desynchronization, the work of art does not resonate with our memories. At complete synchronization, the work resonates with our lives, but cannot add anything. We have heard it before; seen it before. Desynchronization can however lead to activity evoking new context. This corresponds to Danto's “ellipsis”—an empty space to be filled by contextual information. It adds something. It is a mind opener. This implies a change of the internal state of the beholder—a change of the “mindset”—to use a therapeutic term. The “mindset” is understood as a set of activated cognitive procedures in a preactional phase (Gollwitzer and Keller, 2016). I will develop this theme, and argue that the change of the mindset is an art effect and that this effect is relevant to heath.

The mismatch can be experienced as a surprise. The reset leads to an insight: “Oh, what happened now? What a move! This cannot be Charlie Parker; it must be John Coltrane.” Or: “This cannot be Jonas Fisch; it must be Jackson Pollock.” From there on, the beholder puts on her “Coltrane ears” or her “Pollock glasses”. She “tunes in” to the style.

We abandoned the concept of beauty, as an aesthetic concept in the discussion of modernism, but there is a sense of beauty in the “aha”-feeling, when the reset of the contextual stream leads to re-synchronization and the unexpected suddenly fits into a new understanding. This can be experienced in enharmonic modulations (a tone is understood in a new harmonic context) and changes of accents in polyrhythms leading to a new understanding of the rhythm, which is a recurring element in jazz music. On a larger scale, it can be experienced in Messi's unexpected move opening the defense in soccer, in the unforeseen move in chess, leading to the fall of the king, and in Charlie Parker's return to harmony in a solo. Paradoxically, there is in this sense beauty even in Duchamp's fountain, if followed by the aha-experience when the work is connected to art context. However ugly the fountain may seem, there is an elegance in this.

Immanuel Kant labeled the aesthetic experience of awe “the sublime” (Kant, 1951). “Sublime” is Latin meaning “under the limit” (i.e., under the limit of the imaginable). It is a word for an overwhelming experience beyond comprehension. Kant's understanding of the sublime connects with Danto's and Levinson's contextual approach to art in the sense that art challenges presumptions. This art effect is therapeutically interesting, since it entails that the experience of art implies a change of the mindset of the patient. A profound art experience entails that we perceive the world in a new way. It can be operationalized, since desynchronization is biomarker of conflicting information. The framework suggests that the effect can be assessed as a leveling out of ERP indicating a reset of endogenous oscillation.

In three consecutive studies (Liu et al., 2021a,b,c), shed light over the interplay of the mindset, musically induced emotions and synchronization/desynchronization in coping with incongruent information.

These studies show:

1. That meditation (mindfulness) decreases negative emotions caused by sad music and promotes positive emotions of calm music.
2. That meditation improves the capacity to detect emotions in faces as well as in music. The authors found that this behavioral result was accompanied with a leveling out of ERP mismatch reactions to music and faces.
3. That coping with conflicting information, is facilitated by calm as well as happy music. Here, ERP mismatch reactions were stronger if the subjects had listened to sad music and leveled out by calm or happy music.

These findings demonstrate interdependences of meditation, music and conflicting information. The common factor is neural oscillation. The connection between music and neural oscillation has already been accounted in the presentation of Neural Resonance Theory, as has the connection between mismatches and neural oscillation in ERP. When it comes to meditation, the EEC effects (as well as the ERP effect) is established (Cahn and Polich, 2006). This suggests that the interdependence is a resonance effect.

These studies demonstrate that meditation as well as calm and happy music facilitates the reset of endogenous activity. It makes us more flexible. Meditation as well as calm and happy music implies synchronization (because of the absence of mismatches). It improves the ability to cope with conflicts. As stated above with reference to (Cirelli et al., 2014), synchronization facilitates adaptation and cooperation. Taken together Liu's and Cirelli's studies underline the connection between behavioral aspects of empathy and the neurological aspect of synchronization. Synchronization with music implies synchronization with the artist performing the music. This is to say that empathy, can be operationalized as synchronized inter-brain activity measured with EEG hyper-scanning techniques allowing to follow performer/beholder dyads (Peng et al., 2021).

What is the implication to health? As claimed, mismatches are arousing. Several recent studies examine aspects of stress and desynchronization (Jena, 2015; Thome et al., 2017; Lebedeva et al., 2019; Tumati et al., 2021). This concerns inter-individual and intra-individual desynchronization, as well as desynchronization between the brain and the autonomous nervous system. Coping with conflicts is essential for stress management (Fothergill et al., 2004). The most obvious psychosomatic health issue is stress-induced illness. Stress is associated with a row of diseases, including mental illnesses (anxiety, depression, burn out), as well as any disease following from stress impaired immune systems (Morey et al., 2015). Stress is also associated with various heart problems (Stansfeld and Marmot, 2002). This concerns long-term stress, as well as acute stress. In the latter case, this is associated with the “Takotsubo Syndrome”, sometimes tellingly referred to as “The Broken Heart Syndrome”. This is a state reminding of a myocardial infarction. It is possible that desynchronization, long-term as well as acute, following from conflicting information, disturbs the fine-tuned oscillatory neural activity coordinating the activity of the heart.

To sum up: In temporal art, the performer, as well as the beholder, pre-takes the work leading to a synchronized or “quasi-simultaneous” (in Schütz's terminology) co-performance. The pre-takes are implicitly or explicitly acted out: *co-performed*. The motor component in this activity is linked to personal memories. This gives art an *existential dimension*. It resonates with our lives. Co-performed, art can affect us, just as any lived activity can. Life can affect us in any direction. It causes stress, calm, loss of self-confidence, pride, loneliness, togetherness, loss of meaning, new meaning, love, and despair, just to point out a few health relevant mental states. So can art. Surprising and overwhelming (sublime) experiences of art demand resynchronization—a reset of context—a new understanding.

When it comes to the question of operationalization of concepts, the presented framework suggests:

1. That the relation between the body and the mind in psychosomatic medicine can be assessed as intra-brain synchronization/desynchronization (since stress has been connected to desynchronization).
2. That art (as a system) can be studied as synchronized/desynchronized inter-brain activity measured with EEG hyper-scanning techniques allowing to follow performer/beholder dyads.
3. That the art effect can be operationalized as an ERP mismatch, followed by a leveling out of ERP), indicating resynchronization.

Why art? If the point with art therapy is physical activation through embodied perception, we need not go to art. We could just exercise. If the point is that art stimulates us the same way as life does, we do not need art. We should change our lifestyle.

There are five answers to this:

1. Art can be administrated to people who are impaired (who cannot exercise and who are restrained from everyday living due to physical inability, age, dementia, depression, loneliness, sickness, pandemics etc).
2. Art can be designed or selected to individualize therapeutic treatment.
3. The enaction of art can be administrated collectively, promoting shared experiences and social health.
4. The point is not to promote health by physical activation, but that the beholder lives the work of art with her whole being including cognition and emotion.
5. Art challenges presumptions and causes change. The art effect is existential. The challenge for the therapist is to make this happen.

I will use the remaining space of this discussion to expand on the existential dimension. The idea to bring about change by making people participate in a script of movements is traditionally practiced in rituals. The kinship between arts and rituals has been highlighted (Brown and Dissanayake, 2018). As the authors point out, the ritual has many art ingredients: masks, body decorations and insignia. More important, the ritual is a rule-governed sequence of actions and the practice is symbolistic (Brown, 1991). In a ritual, an idea, a fantasy is acted out by the participants following a script of actions. By the collective enactment (explicit or implicit co-performance) of the script, a shared belief system is conjured (since action and perception is a mutual dependency). It becomes lived and felt:

- It is not until the priest repeats “the words of Christ” in the communion and metaphorically serves the Savior's blood and body, that believers feel the presence of the divine.
- It is not until the priest spreads dust over the coffin and utters the very same words from generation to generation: *We therefore commit this body to the ground, earth to earth, ashes to ashes, dust to dust…* that we feel real grief. The exact script unites the mourners with their ancestors, mourning their deceased fathers and mothers, generation after generation. Our human experience and destiny—is synthesized, in the ritual. The pre-take of the ritual actualizes acute loss and acute helplessness.
- It is not until the winner in sports is standing on the podium, listening to the national anthem, that her (and perhaps our) tears start flowing.
- It is not until we sing Christmas carols together, that we feel Christmas in our hearts.

Brown and Dissanayake point out art elements in the ritual. This statement can be reversed: Temporal art has ritualistic elements. The participants pre-take a script of actions. Rituals have traditionally been used to change mental states, such as the conception of the self, and the conception of a life situation, and the conception of destiny. In traditional medicine, the priest and the medic is the same person, guiding individuals to change through the rite. The effect can be dramatic (Bell, 1990). The ritual is a bodily enactment of a shared belief—an imagination. Anderson's statement, “…thinking, perceiving, and acting are synchronous and co-determining” (Anderson, 2016), entails that action determines cognitive activity. I suggest that the enactment of an imagination in a ritual enforces the imagination into imagery—a perceived and lived imagination. This means that the enactment of a work of art, which is an enactment of the imagination of the artist, can change cognitive activity. A profound art experience can change the perspective of the beholder. It can change the mindset.

Why art? This article is the long answer. Condensed, the embodied enactive approach to art suggests:

1. Art is a meeting of imaginations. This meeting is an extended cognitive system comprising the artist, the work and the beholder.
2. Implicit or explicit co-performance (enactment) of the work of art can bring about a change of the mindset. Enacting a work of art is to assimilate the artist's view of life—her imagination.
3. Concerning health, we already know that the mindset is powerful, since the placebo effect is such a huge problem in the testing of new drugs. The force is strong enough to obscure the effect of any salable drug. Although this effect is well researched, it has been considered a confounding variable in medical research. The positive effect of the change of the mindset is beginning to attract attention with strategies such as “mindfulness”, art therapy as well as combinations thereof.

To apply enactive embodiment in art therapy is a challenge for practicing therapists. Enactive embodied theory suggests:

1. *Contextual knowledge is crucial*. Art forms such as abstract art or atonal music has no effect if the patient is unfamiliar with the art form.
2. *Activity is more effective than inactivity*. The framework suggest activity aiming to bring about cognitive change. Explicit co-performance has stronger effects on cognition than implicit co-performance, since it requires a wider activation of motor networks and thus has a stronger impact on embodied memories. Dancing, playing music and choir singing should therefore be more effective than pure music listening. Dancing combines the emotional effect of music with explicit synchronization, socialization and physical exercise. Choir singing synchronizes the participants' explicit motor activity as well as physiological effectors coupled to the activity. The treatment of stress related disorders should not be floating in a dark water tank in order to relax (as has been practiced here and there to cure “burn-out”). This isolates the patient and leaves free space for kneading and anxiety. The choice is not even necessarily calm music. The research of Liu et al. provides a basis for testing happy music as well, since both types facilitate coping with conflicts. Furthermore, daily activity promotes nightly inactivity (sleep).
3. *Collective co-performance* of art leads to shared perspectives, which helps people to get out of personal kneading and anxiety. It initiates a change of perspective. A change from the own perspective, to a shared perspective. It is a possibility to experience that we are not alone with our problems—a possibility to distance ourselves from ourselves.
4. *Temporal art forms* invite collective participation leading to synchronization with the artist and other participants. This points to rhythmic entrainment and suggests that music or combinations with music such as music and respiration, choir singing, and dance should be considered. Music can be composed to guide slow and deep breathing in order to stimulate heart rate variability, which has a parasympathetic (i.e. soothing) effect (Grossman and Svebak, 1987). Singing has a similar effect since singing demands deep and slow respiration (Vickhoff et al., 2013). In choir singing, reported social health effects are not just caused by the fact that choir singing is a social activity. It is also synchronizing the participants at all levels, which has an empathetic social effect. Multimodal stimulation engages larger brain networks. This suggests combinations engaging several senses (e.g., music and moving pictures, music and drama, moving to music).
5. The cognitive-emotional-action interdependency, suggests that not just action, but also emotional interventions can be used in therapy. The fact that music affects people pre-consciously in terms of emotion enables the therapist to set a favorable (positive and relaxed) atmosphere for therapeutic treatment. As we have seen, this promotes coping with conflicts. In addition, it enables the therapist to associate cognitive information with positive feelings (e.g., music and lyrics; music and guided meditation).

Why is it, that people in western societies, so rich on the material side, do not feel well? Why is it, that we with our advanced medical care systems have alarming psychosomatic issues? Why is it, that our children have all these diagnoses, previously unheard of? The answer is not just the inflation of new diagnoses, but the rapid change in our culture. In the online society, we are constantly exposed to news, surprises and conflicts. It has irresistible advantages, but we are not adapted to it?

The reciprocal body-mind connection is well known in psychosomatic medicine. Still, it rarely appears to inform medical practice. In the hospital, we are still “a knee in room four”, “a stomach in room six”, or “a kidney in room three”. De-individualized. Reduced to body parts.

Medical science is aware of this challenge. There is a need of new strategies. Art therapy is an interesting approach. Fancourt and Finn nail evidence of the art effect as the main issue of the field. This hypothesis article accounts for an enactive, embodied understanding of art, suggesting operational definitions of key concepts, including of the art-effect. It points to neural synchronization/desynchronization as the link between the body and the mind. This gives the behavioral aspects of harmony and discord direct operational understandings.

To practice art therapy requires not just an evidence-based strategy; it also demands empathy, creativity, sensitivity, imagination, and skill. It is in itself an art form.

## Data availability statement

The original contributions presented in the study are included in the article/supplementary material, further inquiries can be directed to the corresponding author.

## Author contributions

The author confirms being the sole contributor of this work and has approved it for publication.

## Footnotes

^1^ https://www.youtube.com/watch?v=SRqqiSepLhY
